# Arcuate and Preoptic Kisspeptin Neurons Exhibit Differential Projections to Hypothalamic Nuclei and Exert Opposite Postsynaptic Effects on Hypothalamic Paraventricular and Dorsomedial Nuclei in the Female Mouse

**DOI:** 10.1523/ENEURO.0093-21.2021

**Published:** 2021-08-05

**Authors:** Todd L. Stincic, Jian Qiu, Ashley M. Connors, Martin J. Kelly, Oline K. Rønnekleiv

**Affiliations:** 1 Department of Chemical Physiology and Biochemistry, Oregon Health and Science University, Portland, OR 97239; 2 Division of Neuroscience, Oregon National Primate Research Center, Oregon Health and Science University, Beaverton, OR 97006

**Keywords:** homeostasis, neural circuitry, optogenetics, preautonomic, reproduction, viral tract tracing

## Abstract

Kisspeptin (Kiss1) neurons provide indispensable excitatory input to gonadotropin-releasing hormone (GnRH) neurons, which is important for the coordinated release of gonadotropins, estrous cyclicity and ovulation. However, Kiss1 neurons also send projections to many other brain regions within and outside the hypothalamus. Two different populations of Kiss1 neurons, one in the arcuate nucleus (Kiss1^ARH^) and another in the anteroventral periventricular nucleus (AVPV) and periventricular nucleus (PeN; Kiss1^AVPV/PeN^) of the hypothalamus are differentially regulated by ovarian steroids, and are believed to form direct contacts with GnRH neurons as well as other neurons. To investigate the projection fields from Kiss1^AVPV/PeN^ and Kiss1^ARH^ neurons in female mice, we used anterograde projection analysis, and channelrhodopsin-assisted circuit mapping (CRACM) to explore their functional input to select target neurons within the paraventricular (PVH) and dorsomedial (DMH) hypothalamus, key preautonomic nuclei. Cre-dependent viral (AAV1-DIO-ChR2 mCherry) vectors were injected into the brain to label the two Kiss1 neuronal populations. Immunocytochemistry (ICC) for mCherry and neuropeptides combined with confocal microscopy was used to determine the projection-fields of both Kiss1 neuronal groups. Whole-cell electrophysiology and optogenetics were used to elucidate the functional input to the PVH and DMH. Our analysis revealed many common but also several clearly separate projection fields between the two different populations of Kiss1 neurons. In addition, optogenetic stimulation of Kiss1 projections to PVH prodynorphin, Vglut2 and DMH CART-expressing neurons, revealed excitatory glutamatergic input from Kiss1^ARH^ neurons and inhibitory GABAergic input from Kiss1^AVPV/PeN^ neurons. Therefore, these steroid-sensitive Kiss1 neuronal groups can differentially control the excitability of target neurons to coordinate autonomic functions with reproduction.

## Significance Statement

Hypothalamic kisspeptin (Kiss1) neurons are the most gonadal steroid-sensitive neurons in the brain, and its principal neurotransmitter kisspeptin is essential for sexual development and reproduction through direct excitation of gonadotropin-releasing hormone (GnRH) neurons. Kiss1 neurons also co-express either the classical neurotransmitters GABA, which we document is released only by Kiss1^AVPV/PeN^ neurons or glutamate, which is released by Kiss1^ARH^ neurons. Consequently, Kiss1^AVPV/PeN^ neurons have direct inhibitory and Kiss1^ARH^ neurons direct excitatory actions onto PVH and DMH neurons known to control food intake and energy expenditure, respectively. Therefore, we have found that Kiss1 neurons have a significant input to “preautonomic” neurons known to regulate multiple homeostatic functions, which would help coordinate reproduction with these other functions that are vital for survival of the species.

## Introduction

Early in this century, it was discovered that that mutations in an orphan receptor GPR54 caused hypothalamic hypogonadism in humans, and deletion of GPR54 in mice resulted in defective sexual development and reproductive failure (De Roux et al., 2003; Seminara et al., 2003; d’Anglemont de Tassigny et al., 2007). It was subsequently discovered that a fragment of the peptide metastin, kisspeptin (Kiss1), was expressed in the hypothalamus and deletion of Kiss1 also caused hypothalamic hypogonadism (d’Anglemont de Tassigny et al., 2007). Deletion of GPR54 also results in obesity in female mice (Tolson et al., 2014). The distribution and gonadal steroid regulation of hypothalamic Kiss1 neurons was quickly determined in numerous species including human for review see (Smith, 2008; Oakley et al., 2009; Lehman et al., 2013; Hrabovszky, 2014). Neurons expressing Kiss1 are located primarily in two distinct areas of the forebrain: the preoptic area (POA) and the basal hypothalamus. The preoptic Kiss1 populations in rodents are located in the anteroventral periventricular nucleus (AVPV; Kiss1^AVPV^) and adjacent periventricular nucleus (PeN; Kiss1^PeN^), and these neurons co-express tyrosine hydroxylase (TH), vesicular GABA transporter (vGat), and in some animal models also vesicular glutamate transporter-2 (vGlut2; Cravo et al., 2011; Zhang et al., 2013). Importantly, these rostral Kiss1^AVPV/PeN^ neurons are positively regulated by 17β-estradiol (E2; Smith et al., 2005; Zhang et al., 2013).

The basal hypothalamic Kiss1 population is located in the arcuate nucleus of the hypothalamus (ARH; Kiss1^ARH^) with scattered neurons also in the hypothalamic dorsomedial nucleus (DMH; Clarkson et al., 2009; Bosch et al., 2012). The mRNA expression of the neuropeptides, including Kiss1, NKB (tac2) and prodynorphin (pDyn) within the Kiss1^ARH^ neurons are all inhibited by E2, whereas vGlut2 mRNA, also expressed in Kiss1^ARH^ neurons, and glutamate release are increased by E2 in females (Navarro et al., 2009; Qiu et al., 2018). The Kiss1^AVPV/PeN^ neurons send direct projections onto gonadotropin-releasing hormone (GnRH) neurons and are essential for positive feedback regulation of GnRH and luteinizing hormone secretion (Clarkson et al., 2008; Yip et al., 2015; Qiu et al., 2016; Piet et al., 2018). Kiss1^ARH^ neurons in mice do not appear to contact GnRH cell bodies, but form close anatomic contacts with distal GnRH nerve processes and also exhibit neurophysiological (functional) interaction with Kiss1^AVPV/PeN^ neurons (Yip et al., 2015; Qiu et al., 2016). Later it was discovered that Kiss1^ARH^ neurons project to Proopiomelanocortin and neuropeptide Y/agouti-related peptide (NPY/AgRP) neurons, which suggested that Kiss1 neurons may also be involved in regulating feeding behaviors (Qiu et al., 2018; Padilla et al., 2019). Kisspeptin immunoreactive fibers are located in many different brain regions including median preoptic nucleus (MnPO), ventral lateral septum nucleus (LSV), bed nucleus of the stria terminalis (BST), paraventricular nucleus of the hypothalamus (PVH), supraoptic nucleus (SON), lateral hypothalamus (LH) and DMH, suggesting widespread projections of Kiss1 neurons (Clarkson et al., 2009; Marraudino et al., 2017). However, with the exception of Kiss1 input to GnRH neurons using the Cravo mouse model (Yip et al., 2015), the origins of these fibers are for the most part unknown. In order to study the anatomy and physiology of the two hypothalamic Kiss1 neuronal groups, various animal models expressing Kiss^Cre^ or Kiss^Cre:GFP^ have been produced (Mayer et al., 2010; Cravo et al., 2011; Gottsch et al., 2011; Yeo et al., 2016; Padilla et al., 2018). These Kiss1^Cre^/Kiss1^Cre:GFP^ models differ in several aspects including degree of ectopic expression and in terms of which classical neurotransmitters they express. Therefore, we used the mouse model produced by Padilla and co-workers (Padilla et al., 2018), which was found to exhibit little or no ectopic expression (see Materials and Methods). We did anterograde projection analysis, and channelrhodopsin-assisted circuit mapping (CRACM) to explore their functional input to select target neurons within PVH and DMH, key preautonomic nuclei. These experiments revealed many common, but also several clearly separate projection fields between the two different groups of Kiss1 neurons. In addition, activation of Kiss1 projections to PVH and DMH neurons, revealed excitatory glutamatergic input from Kiss1^ARH^ neurons and inhibitory GABAergic input from Kiss1^AVPV/PeN^ neurons, an indication that these neuronal group can differentially impact target neurons including those in the PVH and DMH.

## Materials and Methods

### Mice

All procedures conducted with animals were according to the National Institutes of Health *Guide for the Care and Use of Laboratory Animals* with approval for all of the animal use procedures from the Oregon Health and Science University (OHSU) Animal Care and Use Committee.

*Kiss1^Cre:GFP^* version 1 (V1; Gottsch et al., 2011), but primarily Kiss1^Cre^ version 2 (V2; Padilla et al., 2018), female mice were used in these experiments. In addition, Kiss1^Cre^::GnRH^GFP^ and Kiss1^Cre^::Ai32 mice were produced by crossing heterozygous Kiss1^Cre^ V2 female mice with GnRH^GFP^ mice (Suter et al., 2000) or with the reporter line Ai32 mice (Jackson, Stock No. 024109), respectively. It should be noted that Kiss1^Cre^ V2 mice when crossed with a conditional reporter line, V2 mice express Kiss1 neurons in the different brain nuclei with little or no ectopic expression (Padilla et al., 2018). The animals were housed under constant temperature (21–23°C) and 12/12 h light/dark cycle schedule (lights on at 6 A.M. and lights off at 6 P.M.), with free access to food (Lab Diets 5L0D) and water. Where specified, the Kiss1*^Cre^*V2 mice or Kiss1^Cre^::GnRH^GFP^ mice received viral injections to express channelrhodopsin 2 (ChR2)-mCherry in Kiss1 neurons (see AAV delivery below).

*Kiss1^Cre:GFP^* animals used in the current study have been documented to express *Kiss1*, *NKB*, *pDyn*, and *Slc17a6* (*Vglut2*) mRNAs in Kiss1^ARH^ neurons and has been found to release kisspeptin, NKB and glutamate on activation (Gottsch et al., 2011; Qiu et al., 2018). Importantly, this animal model does not express *Slc32a1* (*vGAT*) or release GABA from Kiss1^ARH^ neurons (Nestor et al., 2016; Qiu et al., 2016, 2018). The Kiss1^AVPV/PeN^ neurons in the same animal model have been documented to express *Kiss1*, *TH*, and *Slc32a1* (*Vgat*) mRNAs and found to release kisspeptin and GABA when activated, but do not express (*Slc17a6*) *Vglut2* mRNA or release glutamate (Zhang et al., 2013, 2015; Qiu et al., 2016). Given that Kiss1^AVPV/PeN^ neurons in other Kiss1^Cre^ animals have been found to co-express *Slc32a1* and also *Slc17a6* mRNAs (Cravo et al., 2011), we have included studies to further document the diverse expression of *Vgat* and *Vglut2* in the two populations of hypothalamic Kiss1 neurons in the animal models used in the current study (Gottsch et al., 2011; Padilla et al., 2018).

### AAV delivery

Only Kiss1^Cre^ (V2) animals were used for Cre-dependent ChR2-mCherry injections. Fourteen to 21 d before each experiment, Kiss1^Cre^, and Kiss1*^Cre^::*GnRH*^GFP^* male or female mice (>60 d old) received bilateral AVPV/PeN or ARH injections of a Cre-dependent adeno-associated viral (AAV; serotype 1) vector encoding ChR2 fused to mCherry fluorescent protein (AAV-EF1α-DIO-ChR2:mCh). Using aseptic techniques, anesthetized mice (1.5% isoflurane/O_2_) were placed in a Kopf stereotaxic apparatus and received a medial skin incision to expose the surface of the skull. Once bregma was identified, the Kopf stereotaxic alignment tool (Model 1905) was used to level the head (*y*: −1.200). Next, the bregma to λ distance was measured and compared with the expected size of 4.21 mm. The final *y*-coordinates were adjusted to account for deviations from this average length. For ARH injections, two holes were drilled into the skull at designated coordinates from bregma (*x*: ±0.33 mm; y: –1.185 mm). In order to deliver the virus, a glass micropipette (Drummond Scientific #3-000-203-G/X) was fabricated with a Narishige PE-2 puller and beveled (tip diameter = 45 μm), back filled with mineral oil and front loaded with an aliquot of AAV using a Nanoject II (Drummond Scientific). The pipette tip was positioned at *x*: –0.33 mm lateral; *y*: –1.185 mm and lowered to z = –5.800 mm (surface of brain z = 0.0 mm). Of note, the Kiss1^Cre^ (V2) x GnRH mice typically had a larger bregma to λ distance and standard *y*-coordinates were often scaled up 5–15%. Next, the AAV (2.0 × 10^12^ particles/ml) was injected at a rate of 100 nl/min (300 nl total), raised to −5.70 mm for a second injection (200 nl total) and then left in place for 10 min postinjection. Then, the pipette was slowly removed from the brain, pausing briefly at −5.5, −5.0, and finally −4.5 mm. The pipette was then fully retracted and moved to the other hemisphere, now x = +0.33 mm, and the process was repeated. The skin incision was closed using VetaBond (3 M) skin adhesive, and each mouse received analgesia (carprofen; 5 mg/kg, sc). For AVPV/PeN injections the same leveling and measurement steps were taken, and the coordinates were *x*: ±0.33, *y*: 0.55, *z*: −5.1 and −4.7. To better cover the longer and thinner shape of the AVPV/PeN, the virus was injected at two sites separated by 0.5 mm (300 nl/site, 100 nl/min). Unlike the ARH, the pipette was left in place for 10 min after injection at each site to allow for even distribution of the virus and to limit viral reflux as the pipette was withdrawn from the brain.

### Ovariectomy (OVX) and estradiol treatment

When necessary, at least 7 d before each experiment, ovaries were removed while under isoflurane inhalation anesthesia (Piramal Enterprises Limited). Each mouse received analgesia (carprofen; 5 mg/kg, sc) on the day of operation. E2 benzoate (E2B) treatments were as follows. Each animal was injected on days 4–5 following OVX with 50 μl of 0.25 μg E2B in sesame oil, followed on day 6 with 1.50 μg E2B and used for experiments on day 7. High-circulating (proestrous) levels of E2 were verified by the uterine weights (>100 mg) at the time of death. Other OVX animals did not receive E2B, but equal volume (50 μl) of sesame oil.

### Immunocytochemistry (ICC)

Female Kiss1^Cre^ mice or Female Kiss1^CreGFP^::GnRH^GFP^ mice, with injection of ChR2-mCherry in the ARH, or AVPV/PeN were prepared for ICC as follows: coronal hypothalamic blocks (2–3 mm each) were fixed by immersion in 4% paraformaldehyde, cryoprotected in 30% sucrose solution, frozen at −55°C, sectioned coronally on a cryostat at 20 μm, and thaw-mounted on Superfrost Plus slides (Thermo Fisher Scientific). Sections were rinsed in PB (0.1 m phosphate buffer, pH 7.4) for at least 30 min. Next, sections were incubated with normal serum corresponding to the host for the secondary antiserum (5% normal serum with 0.3% Triton X-100 in PBS for 30 min), rinsed in PB and then incubated for 48 h at 4°C in rabbit or goat polyclonal antiserum against mCherry (1:10 000; ab167453 Abcam Inc or Biorbyt, respectively). Some sections were double-labeled for mCherry and GFP using combined mCherry (1:10 000; rabbit) and biotinylated GFP (1:5000; goat) antibodies to illustrate co-expression of mCherry in Kiss1^Cre-GFP^ neurons or projections of mCherry/Kiss1 fibers onto GFP-expressing GnRH neurons. Brain sections were also reacted with rabbit polyclonal antisera against kisspeptin, GnRH, oxytocin (OT), or vasopressin using the Caraty kisspeptin antibody (no. 564; 1:2500; Franceschini et al., 2006), GnRH antibody (EL-14; 1:5000; Ellinwood et al., 1985), OT antibody (1:5000; Morris et al., 1980) or arginine vasopressin antibody (AVP; 1:5000; Dave et al., 1985) together with the mCherry antibody produced in goat described above. The specificity of these antisera has been documented previously (Ellinwood et al., 1985; Rønnekleiv et al., 1990; Franceschini et al., 2006). After rinsing, sections stained for mCherry were incubated in goat-antirabbit IgG antibody conjugated to Alexa Fluor 594 (1:500; Jackson ImmunoResearch). Sections stained for dual mCherry and kisspeptin, GnRH, OT, or AVP were first incubated for 2–3 h at room temperature with biotinylated bovine anti goat (1:500) and next with a mixture of streptavidin-Alexa Fluor 594 (1:2500) and goat-antirabbit IgG antibody conjugated to Alexa 488 (1:1000; Jackson ImmunoResearch). Following a final rinse overnight, slides were coverslipped with gelvatol containing the anti-fading agent, 1,4-diazabicyclo(2,2)octane (DABCO; Sigma-Aldrich; Grachev et al., 2016). We also attempted to stain for dynorphin and vGluT2 in the PVH and CART in the DMH to study Kiss1 inputs to these neurons. However, we were not able to consistently detect cell bodies in these brain regions, although we were able to detect dynorphin cells in the ARH, SON, and the striatum. It should be noted that most studies of dynorphin cells in the PVH in mice are based on using Pdyn-Cre animals (Shah et al., 2014). Therefore, we used single-cell RT-PCR (scRT-PCR) to identify *Pdyn*, *Slc17a6* (*Vglut2*), or *Cart* mRNA following whole-cell recording (see below).

### Widefield Imaging

Photomicrographs of GFP or mCherry labeling were initially acquired using a Nikon E800 fluorescent microscope (Eclipse E800; Nikon Instruments) equipped with a fiber illuminator (Intensilight C-HGFI; Nikon Instruments) and a high-definition digital microscope camera head (DS-Fi1; Nikon Instruments) interfaced with a PC-based camera controller (DS-U3; Nikon Instruments).

### Confocal imaging

Confocal micrographs were acquired using one of three Zeiss AxioObserver inverted laser scanning confocal microscopes. The Zeiss LSM 780 was mounted on a motorized stand and equipped with water immersion 20× and 40× (0.8 and 1.2 numerical aperture, respectively) apochromatic objectives. For mCherry/Cy3 or mCherry/Alexa Fluor 594, 561-nm excitation was provided by a DPSS laser and detection was in the 585- to 681-nm range. GFP/Alexa Fluor 488 was excited with a 488 nm Argon laser and detection was in the 502- to 571-nm range. For super-resolution images, we used both a Zeiss LSM 880 and 980 confocal microscope equipped with Fast AiryScan detector. The LSM 880 was equipped with argon and DPSS lasers for 488- and 561-nm excitation, respectively. The LSM 980 was equipped with 488 diode (10 mW) and 561 DPSS lasers (10 mW). For the green channel, bandpass filters for 420–480 and 495–550 nm were used. The red channel used a 570- to 620-nm bandpass and a 645-nm lowpass filter. Both systems used a GaAsP photomultiplier tube for detection, but the LSM 880 used Airyscan 1 (eight channels) and the LSM 980 used Airyscan 2 (32 channels). Following capture, images underwent Airyscan deconvolution in Zen Black/Blue software (Zeiss) to produce super-resolution images (74 nm/pixel) before tiles were stitched together to produce a single Z-stack. Maximum intensity projections and initial Brightness/Contrast adjustments were made in FIJI (ImageJ) before saving a high resolution RGB tif version of the image. Final adjustments and cropping were made in Adobe Photoshop.

### Close contacts

Immunofluorescence was captured using a Zeiss Plan-Apochromat 20 × 0.8 NA air objective, 1.5× zoom, a resolution of 3048 × 3048 per tile, 10% tile overlap, and at a z-increment of 0.3–0.6 μm. Clearly visible immunoreactive green somata in each region were numbered in Adobe Illustrator and the presence of close contacts by (Kiss1) ChR2-mCherry fibers determined. Full Z-stack images were loaded in Zen Software (Zeiss). Close contacts were considered to be present when fibers contoured to cells and proximity was sufficiently close as to leave no intervening black pixels. Even with super-resolution (74 nm effective resolution), synapses cannot be resolved, but close proximity was reflected in the presence of yellow pixels when examining a single focal plane. Sections from at least three different mice underwent confocal analysis for the presence of close contacts. The percentage of close contacts for each animal were used to create a group average and standard error of the mean to give a qualitative assessment of typical innervation (Schindelin et al., 2012). The number of mice per group as well as the total close contacts and cells in each group are listed.

Additional image processing was performed using FIJI (ImageJ), Adobe Photoshop CC (Adobe Systems), and Zen (Zeiss, RRID:SCR_013672) software. Maximum intensity projections of z-stacks containing 10–50 optical images are presented unless otherwise specified. Brightness and contrast have been adjusted to aid in the visualization of Kiss1 neuronal projections.

### Electrophysiology

Having identified Kiss1^AVPV/PeN^ and Kiss1^ARH^ fiber inputs to PVH and DMH neurons, we were interested to explore potential functional interactions. Therefore, Kiss1^Cre^ (V2) animals were used for Cre-dependent ChR2-mCherry injections and subsequent optogenetic analysis of Kiss1 inputs to PVH and DMH neurons. The recordings were done blindly, the cell content harvested following the whole-cell recordings and the cell identified using scRT-PCR (see below). Fourteen to 21 d before each experiment, Kiss1^Cre^ female or male mice (>60 d old) received bilateral AVPV/PeN or ARH injections of a Cre-dependent AAV (serotype 1) vector encoding ChR2 fused to mCherry fluorescent protein (AAV-EF1α-DIO-ChR2:mCh). Coronal brain slices containing the PVH or DMH from gonadectomized E2B or oil-treated AAV-EF1α-DIO-ChR2:mCh-injected Kiss1^Cre^ mice in AVPV/PeN or ARH, respectively, were prepared using established techniques. Whole-cell, patch recordings were performed in voltage clamp and current clamp using an Olympus BX51W1 upright microscope equipped with video-enhanced, infrared-differential interference contrast (IR-DIC) and an Exfo X-Cite 120 Series fluorescence light source. Electrodes were fabricated from borosilicate glass (1.5-mm outer diameter; World Precision Instruments) and filled with a normal internal solution: 128 mm potassium gluconate, 10 mm NaCl, 1 mm MgCl_2_, 11 mm EGTA, 10 mm HEPES, 2 mm ATP, and 0.25 mm GTP (pH was adjusted to 7.3–7.4 with 1N KOH, 290–300 mOsm) or for measurement of IPSCs, patch pipettes were filled with a high chloride solution: 140 mm KCl, 10 mm HEPES, 0.1 mm EGTA, 5 mm MgCl_2_, 0.3 mm Na-GTP, and 5 mm K_2_-ATP, pH was adjusted to 7.3–7.4 with 1N KOH, 290–300 mOsm. Pipette resistances ranged from 3 to 5 MΩ. In whole-cell configuration, access resistance was <20 MΩ; access resistance was 80% compensated. For optogenetic stimulation, a light-induced response was evoked using a light-emitting diode (LED) 470-nm blue light source controlled by a variable 2A driver (ThorLabs) with the light path delivered directly through an Olympus 40× water-immersion lens. LED light pulses were 5–10 ms long and 0.2–0.9 mW at the surface of the tissue. Electrophysiological signals were amplified with an Axopatch 200A and digitized with Digidata 1322A (Molecular Devices, Foster City, CA), and the data were analyzed using p-Clamp software (version 9.2, Molecular Devices). The liquid junction potential was corrected for all data analysis. All drugs used in the electrophysiological experiments were purchased from Tocris Bioscience unless otherwise specified. DL-amino-5-phosphonovaleric acid (AP5; 50 mm), 6-cyano-7-nitroquinoxaline-2, 3-dione (CNQX; 10 mm), CGP55845 (10 mm), and 4-aminopyridine (4-AP; 500 mm) were dissolved in H_2_O. Tetrodotoxin (TTX) was purchased from Alomone Labs (1 mm) and dissolved in H_2_O. Tetraethylammonium chloride (TEA; 7.5) was purchased from Sigma-Aldrich and dissolved in aCSF. Picrotoxin (100 mm) was dissolved in dimethyl sulfoxide (DMSO). Aliquots of the stock solutions were stored as appropriate until needed for experiments.

### scRT-PCR

After electrophysiological recording, the cytosol of recorded cells was harvested and used for *post hoc* identification. Briefly, the recorded cells were harvested with gentle suction to the recording pipette and expelled into a siliconized 0.5-ml microcentrifuge tube containing a solution of 1× Invitrogen Superscript III buffer, 15 U of Rnasin (Promega), 10 mm of dithiothreitol (DTT) and diethylpyrocarbonate (DEPC)-treated water in a total of 5 μl for a single cell. cDNA synthesis was performed on single cells in a 20-μl reaction volume using Superscript II reverse transcriptase (Invitrogen) according to manufacturer’s protocol and stored at −20°C. Controls included water blanks, and hypothalamic tissue RNA reacted with and without reverse transcriptase (-RT). Primers were designed using Clone Manager software (Sci Ed Software) to cross at least one intron-exon boundary and optimized for single-cell mRNA determination following whole-cell recording (see Table 1). scPCR was performed on 3–4 μl of cDNA in a 30 μl reaction volume using GoTaq polymerase (Invitrogen) combined with Taqstart Antibody (Takara) according to manufacturer’s protocol; then amplified 50 cycles using a C1000 Thermal Cycler (Bio-Rad). The PCR product was visualized with ethidium bromide on a 2% agarose gel.

**Table 1:** Primer table

Gene name (encodes for)	Accession number	Primer location (nt)	Product length (bp)	Annealing temperature (°C)
*Kiss1* (Kiss1)^*a*^	NM_178260	64–80	120	57
		167–183		
*Slc32a1* (vGAT)^*a*^	NM_009508	813–834	137	59
		928–949		
*Slc17a6* (vGluT2)^*a*^^,*b*^	NM_080853	1677–1694	136	57
		1795–1812		
*Pdyn* (Dyn)^*b*^	NM_018863	210–228	154	58
		345–363		
*Cart* (CART)^*b*^	NM_013732	288–305	157	59
		427–444		
*Lepr* (LeptRec)^*b*^	NM_146146	3185–3205	112	62
		3276–3296		

a primers used for identifying dispersed single neurons.

b primers used for identifying single neurons after electrophysiological recording.

## Results

### AVPV/PeN injections of ChR2-mCherry

**Figure 1.**
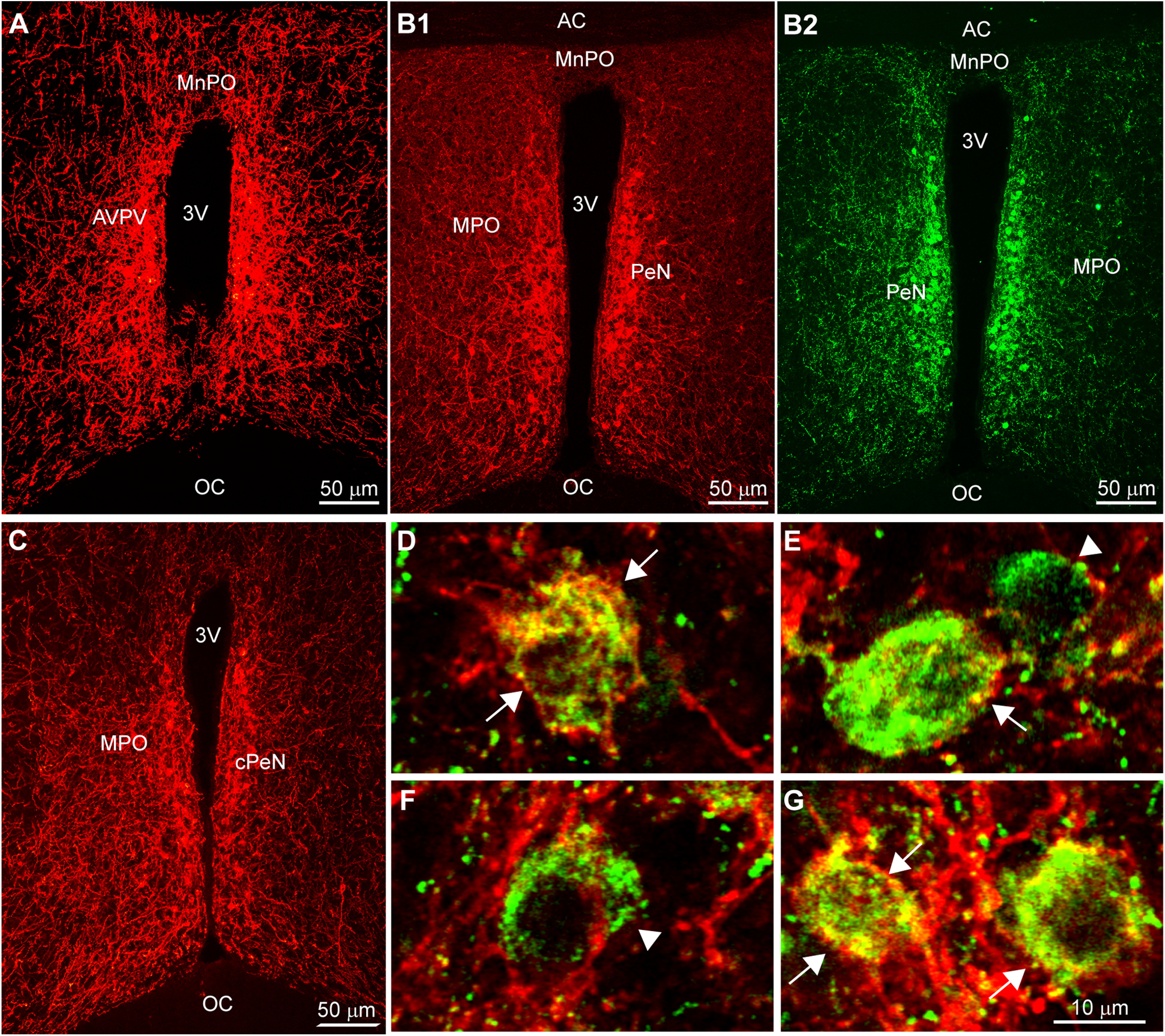
Kiss1^Cre^-dependent ChR2-mCherry expressing neurons in the AVPV-PeN are also positive for kisspeptin. ***A***, ***B1***, ***C***, Low-magnification, fluorescent images of Cre-driven ChR2-mCherry expression enhanced with ICC for mCherry. mCherry-labeled Kiss1 cells are distributed within the AVPV (***A***) and in a more narrow vertical band along the third ventricle (3V) from rostral to caudal PeN (***B1***, ***C***). The section in ***B1*** was also immunostained for kisspeptin (***B2***). ***D–G***, Composite confocal images demonstrating that cells exhibiting Cre-driven mCherry expression are co-labeled by the Caraty kisspeptin antibody (green; arrows), but not all immunoreactive kisspeptin neurons also expressed ChR2-mCherry (arrow-head), likely because of incomplete coverage/infection by injected virus. AC, anterior commissure; AVPV, anteroventral periventricular nucleus; MnPO, median preoptic nucleus; MPO, medial preoptic nucleus; PeN, periventricular nucleus; OC, optic chiasm; 3V, third ventricle). Scale bars: 50 μm (***A–C***) and 10 μm (***D–G***).

**Figure 2.**
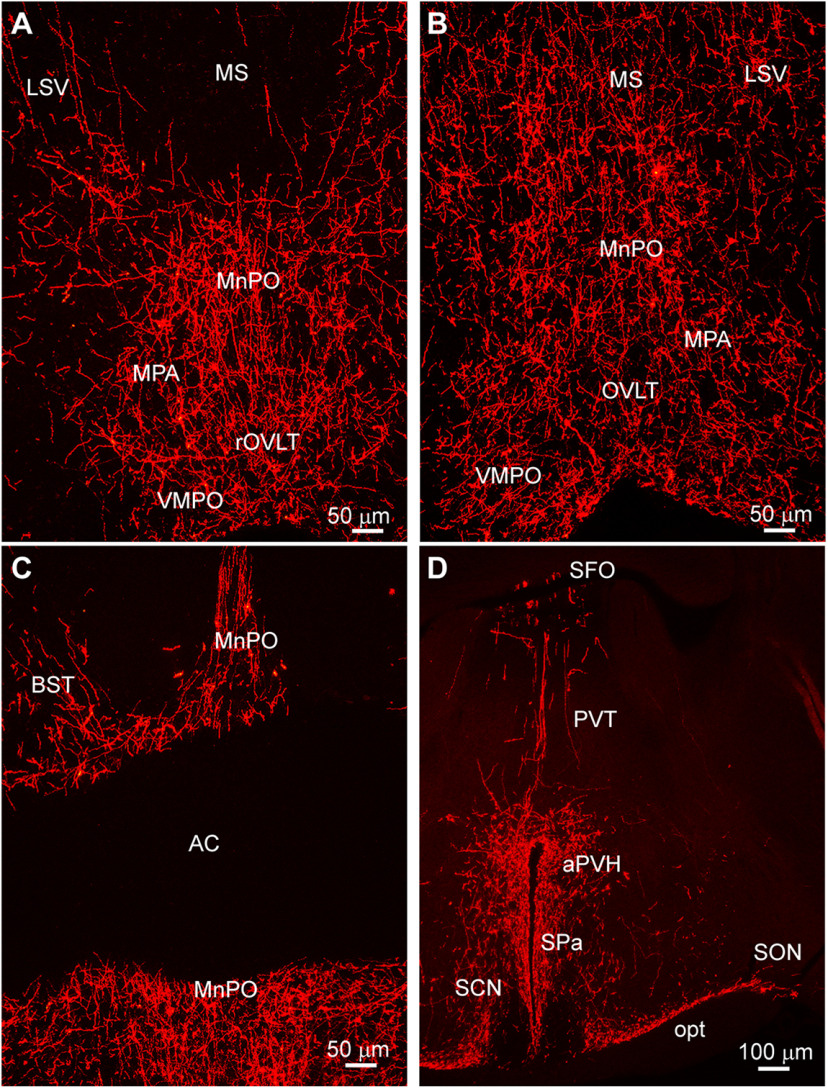
Kiss1^AVPV/PeN^ projections densely innervate different nuclei within the POA-AH. ***A–C***, Representative sections from rostral to caudal POA illustrating Kiss1^Cre^::ChR2-mCherry fiber-projections from POA Kiss1 neurons to different nuclei including the VMPO, MnPO, LSV, and the BST. ***D***, Low-power image of a section through the anterior hypothalamus illustrating Kiss^Cre^::ChR2-mCherry fiber-projections to the aPVH, PVT, SFO, and SON. Note the SCN is essentially devoid of Kiss1^Cre^:ChR2-mCherry fiber-input. LSV, lateral septal nucleus, ventral; MS, medial septal nucleus; MnPO, median preoptic nucleus; MPA, medial POA; VMPO, ventromedial preoptic nucleus; OVLT, organum vasculosum lamina terminalis; BST, bed nucleus stria terminalis; SFO, subfornical organ; PVT, paraventricular nucleus thalamus; aPVH, anterior paraventricular nucleus, hypothalamus; SPa, subparaventricular zone; SCN, suprachiasmatic nucleus; opt, optic tract; SON, supraoptic nucleus. Scale bars: 50 μm (***A*–*C***) and 100 μm (***D***).

We delivered bilateral injections of AAV-Ef1α-DIO-mCherry:ChR2 to the AVPV/PeN of Kiss1^Cre^ (*n* = 5) or Kiss1^Cre:GFP^::GnRH^GFP^ (*n* = 6) female mice, which resulted in labeled Kiss1 cell bodies bilaterally from the AVPV rostrally to the caudal part of the PeN (Fig. 1*A–C*). ICC was used to enhance the Cre-dependent labeling and aid in visualization. In order to confirm specificity of Cre-driven mCherry expression in Kiss1 cells, several tissue sections were also co-reacted with a kisspeptin antibody (Fig. 1*B1*,*B2*; Table 3). While the majority of neurons displayed co-localization of both fluorophores, several neurons were green-only (Fig. 1*E*,*F*). This would indicate that while the AAV/Cre-driven labeling of Kiss1 cells is specific (Nestor et al., 2016), the antibody directed against kisspeptin labeled a greater percentage of the total Kiss1^AVPV/PeN^ population. Additionally, no mCherry-labeled cells were detected in the ARH, indicating that any projections noted in these animals originated from the AVPV/PeN. These Kiss1^AVPV/PeN^ neurons were found to send extensive fiber projections rostrally to various POA regions including the ventromedial preoptic (VMPO) and medial POA (MPA) from rostral to caudal, organum vasculosum of lamina terminalis (OVLT), and the median preoptic nucleus (MnPO; Fig. 2*A–C*). Interestingly, both the VMPO and MnPO are known to be involved in temperature regulation (Tan et al., 2016; Abbott and Saper, 2017).

**Figure 3.**
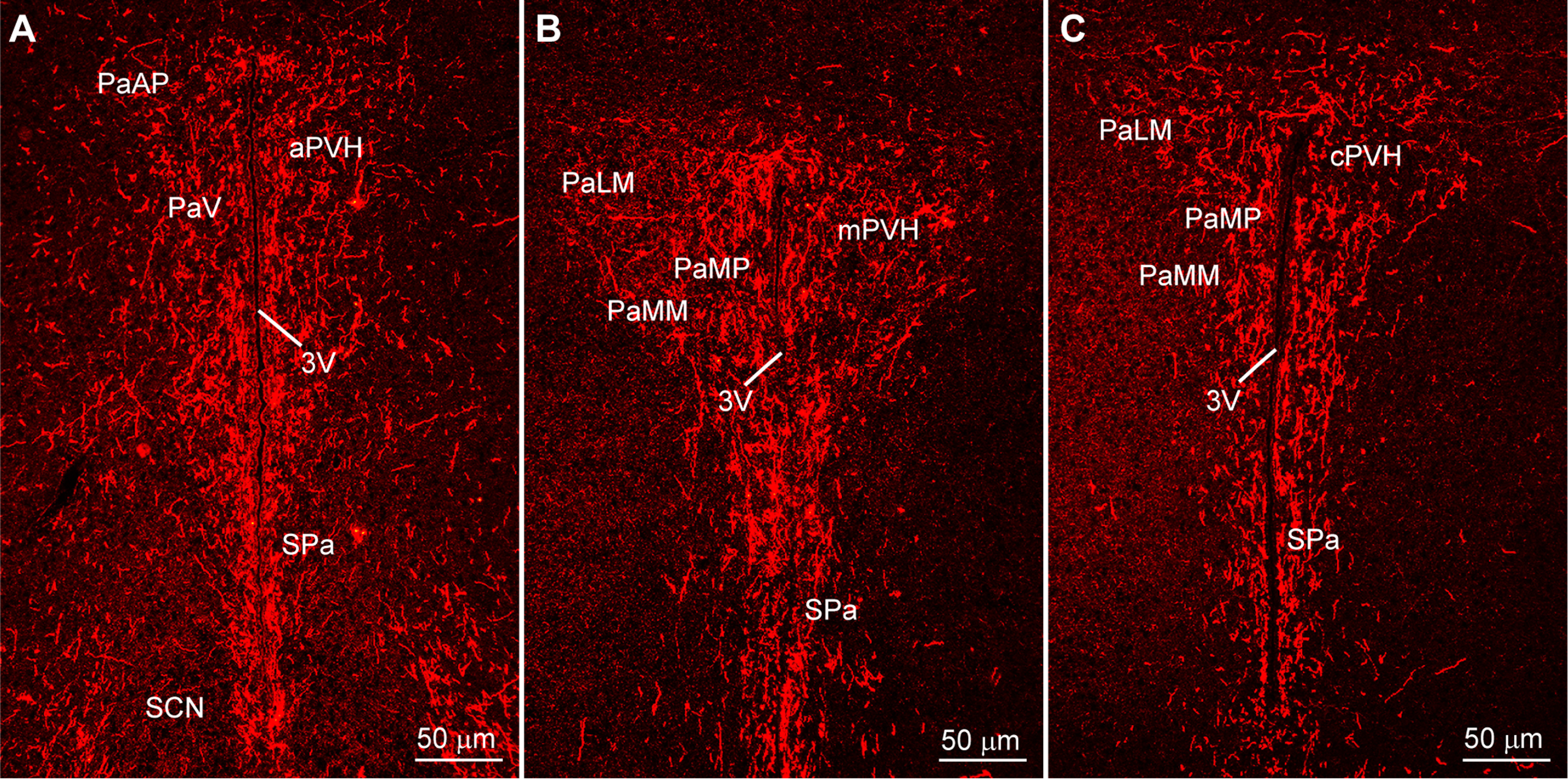
Kiss1^AVPV/PeN^::ChR2-mCherry projections to the PVH. ***A–C***, ChR2-mCherry fluorescent images of the rostral to caudal progression through the anterior hypothalamus showing dense innervation of the PVH by Kiss1^AVPV/PeN^ fibers. Scale bar: 50 μm. PaAP, paraventricular hypothalamus anterior parvicells; PaV, paraventricular hypothalamus ventral; PaLM, paraventriclular hypothalamus lateral magnocells; PaMM, paraventricular hypothalamus medial magnocells; PaMP, paraventricular hypothalamus medial parvicells. For further abbreviations, see legend to Figure 2.

**Figure 4.**
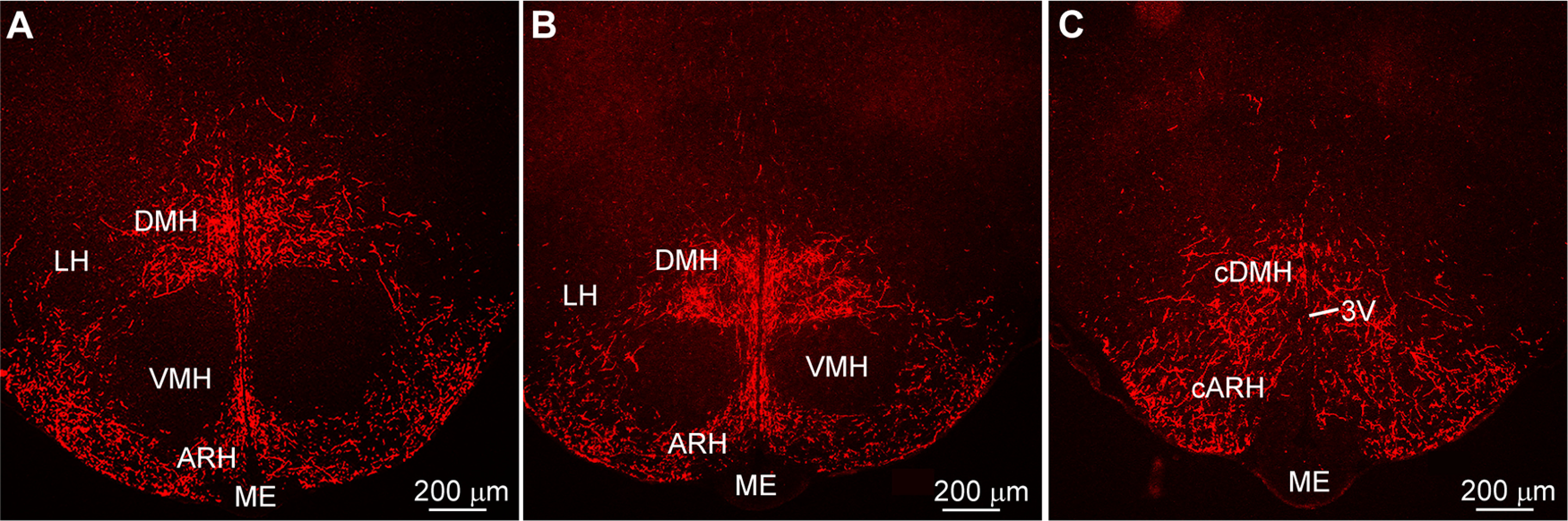
Kiss1^AVPV/PeN:^ChR2-mCherry projections to the DMH. ***A–C***, ChR2-mCherry fluorescent images of the rostral to caudal progression through the basal hypothalamus showing dense innervation of the DMH by Kiss1^AVPV/PeN^ fibers. Note the extensive fiber-projection to the DMH from rostral to caudal, and the ventral fiber-bundle to the ARH, but not the ME or the VMH. Scale bar: 200 μm. DMH, dorsomedial hypothalamic nucleus; LH, lateral hypothalamic area; VMH, ventromedial hypothalamic nucleus; ARH, arcuate hypothalamic nucleus; ME, median eminence.

In addition, Kiss1^AVPV/PeN^::ChR2-mCherry-labeled neuronal fibers extended dorsolaterally toward the ventrolateral septal areas (LSV), medial septum (MS), and bed nucleus stria terminalis (BST; Fig. 2*A–C*). At the level of the suprachiasmatic nucleus (SCN), fibers extended dorsally toward the hypothalamic anterior parvocellular PVH (aPVH) and further dorsal into the paraventricular nucleus of the thalamus (PVT). ChR2-mCherry-labeled fibers were also observed in the subfornical organ (SFO; Fig. 2*D*). Although ChR2-mCherry fibers surrounded the SCN, the SCN itself appeared to lack fiber-input from these rostral Kiss1^AVPV/PeN^ neurons (Fig. 2*D*). The ChR2-mCherry-labeled Kiss1 fibers also extended laterally along the optic tract (opt) as well as close to and, in some cases, into the medial part of the SON (Fig. 2*D*). In addition, fibers originating in the Kiss1^AVPV/PeN^ innervated the PVH and the subparaventricular zone (SPa) from rostral to caudal with the densest projections to the medial parts of the PVH (Fig. 3). Further caudal the fiber-projections were particularly dense in the DMH from rostral to caudal, whereas the VMH was essentially devoid of ChR2-mCherry-labeled Kiss1^AVPV/PeN^ fibers (Fig. 4). ChR2-mCherry fibers also reached parts of the ARH but did not extend into the median eminence (ME; Fig. 4). It should be emphasized that this projection pattern was similar in all the Kiss1^AVPV/PeN^::ChR2-mCherry injected females (*n* = 11).

### ARH injections of ChR2-mCherry

**Figure 5.**
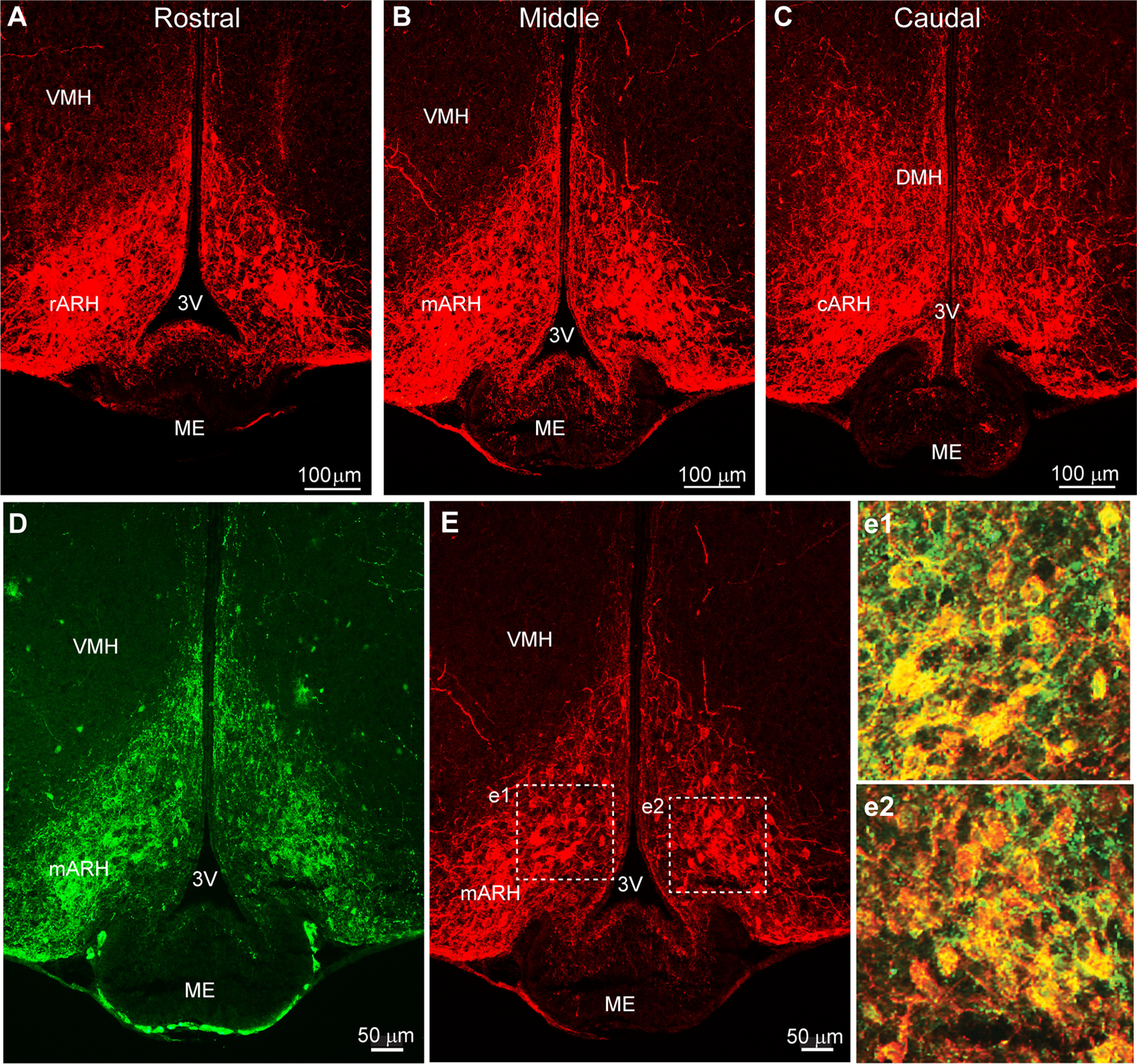
Kiss1^Cre^-dependent ChR2-mCherry expressing neurons in the ARH. ***A–C***, Fluorescent images of the basal hypothalamus displaying Cre-driven expression of ChR2-mCherry-labeled neurons within the arcuate nucleus (ARH) from rostral to caudal. Labeled fibers are primarily located within the internal zone of the ME. The section in ***E*** was also immunostained for kisspeptin (***D***). ***e1***, **e*2***, Composite confocal images demonstrating that cells exhibiting Cre-driven mCherry expression are co-labeled with a kisspeptin antibody. For abbreviations, see legend to Figure 4. Scale bars: 100 μm (***A*–*C***) and 50 μm (***D***, ***E***).

Bilateral injections of Cre-dependent AAV-Ef1α-DIO-mCherry:ChR2 into the ARH of Kiss1^Cre^ (OVX; *n* = 6) or Kiss1^Cre^::GnRH^GFP^ (OVX, *n* = 5) female mice resulted in labeled Kiss1 cell bodies bilaterally throughout the ARH from rostral to caudal (Fig. 5*A–C*). Cell bodies were clearly seen clustered in the more rostral and middle portions of ARH (rARH and mARH). Kiss1 neurons become more scattered in the caudal ARH (cARH) with a small number residing in the DMH (Fig. 5*C*). We found that the majority of ChR2-mCherry labeled ARH neurons were double-labeled for kisspeptin (Fig. 5*D*,*E*). In addition, ChR2-mCherry fibers were observed in the internal zone of the ME, connecting Kiss1^ARH^ neurons on both sides of the ventricle. Kiss1^ARH^ fibers also interact with GnRH fibers in the ME area (see below).

**Figure 6.**
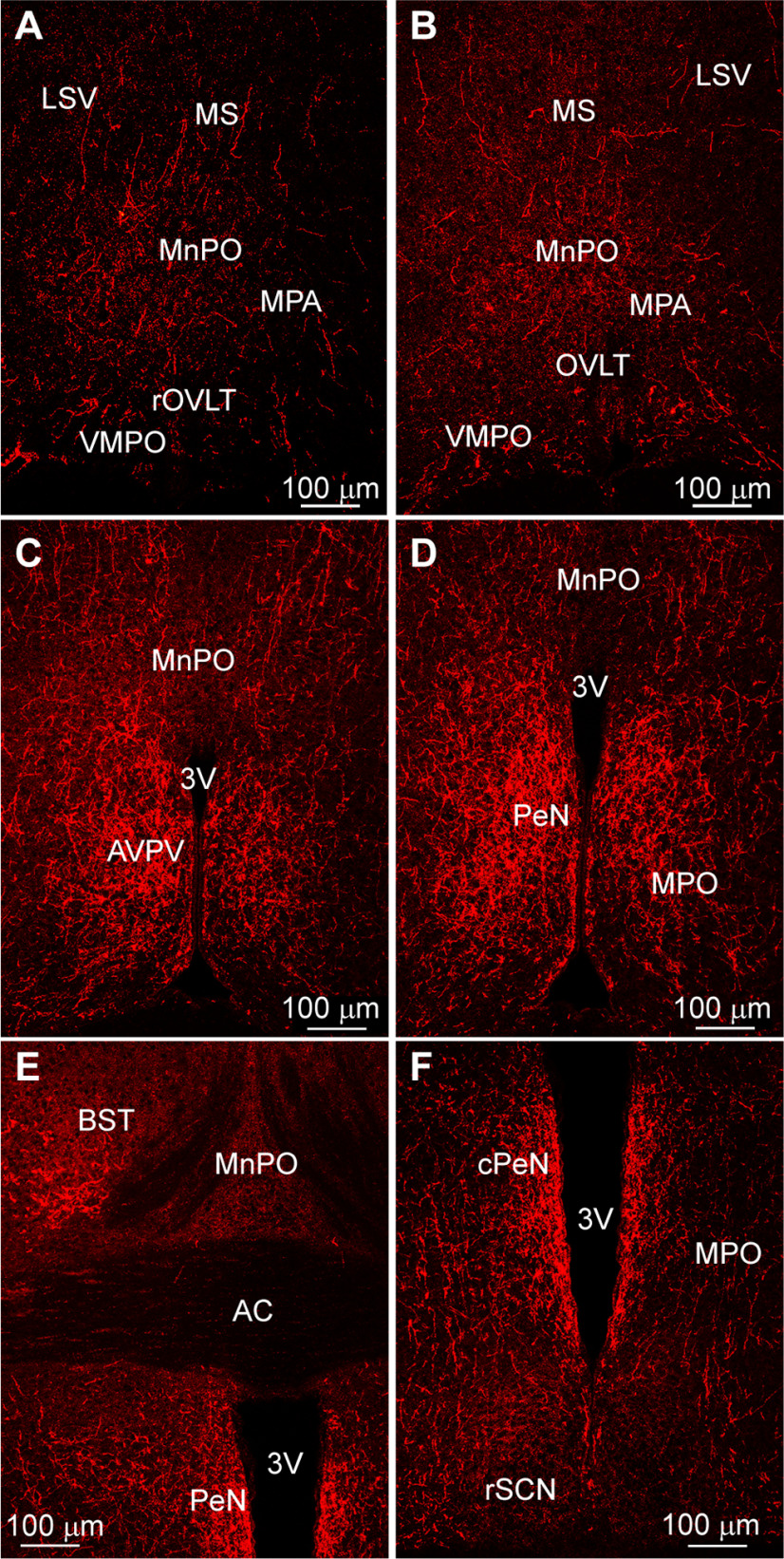
Kiss1^ARH^::ChR2-mCherry projections to the POA. ***A***, ***B***, Fluorescent images of the Cre-driven ChR2-mCherry projections from Kiss1^ARH^ neurons to the rostral POA, a region where most of the GnRH neurons are located. ***C–F***, Kiss1^ARH^ neurons send extensive projections to the AVPV, PeN, MPO, and BST. Note the slight fiber-input to the VMPO, OVLT, MPA, and MnPO, rostral brain regions important for reproduction and temperature regulation. For abbreviations, see legend to Figure 2. Scale bar: 100 μm.

**Figure 7.**
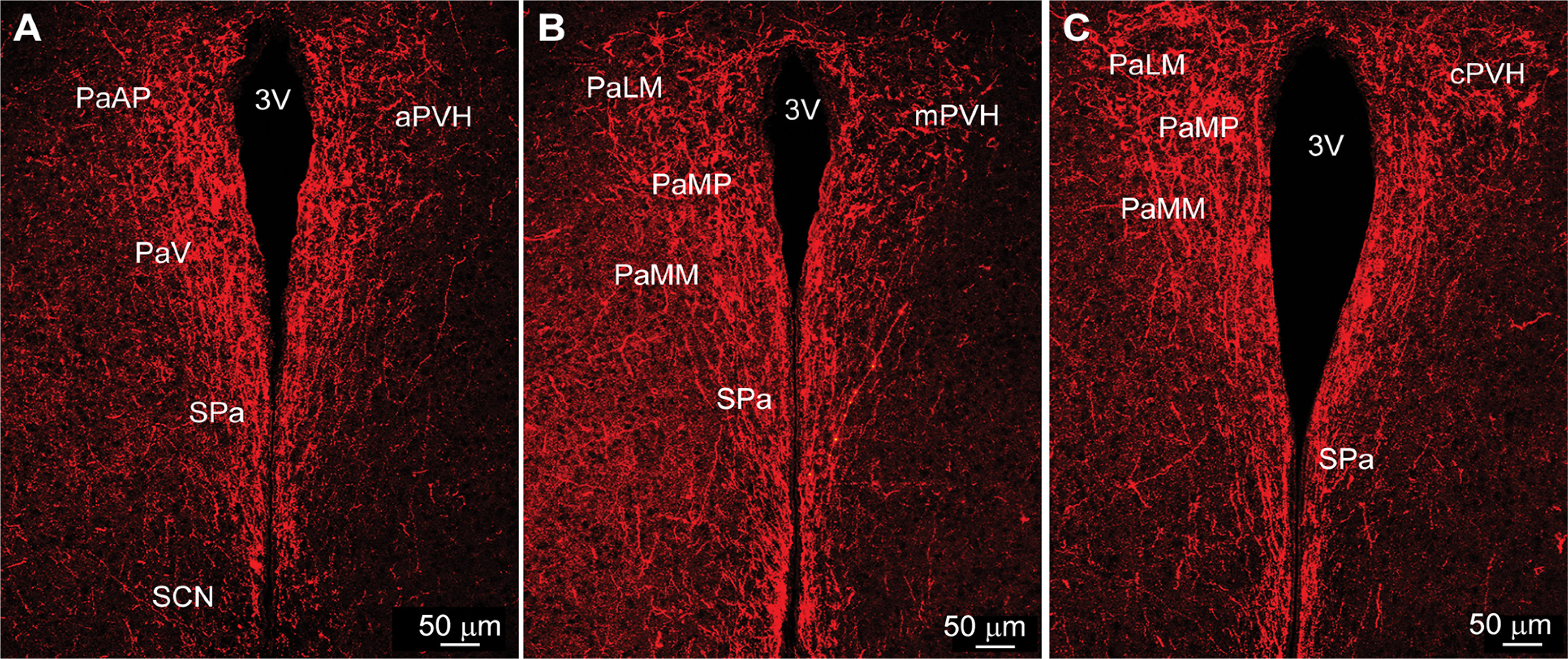
Kiss1^ARH^::ChR2-mCherry projections to the PVH. ***A–C***, ChR2-mCherry fluorescent images of the rostral to caudal progression through the anterior hypothalamus showing dense fiber-input to the PVH by Kiss1^ARH^ fibers. For abbreviations, see legend to Figures 2, 3. Scale bar: 50 μm.

**Figure 8.**
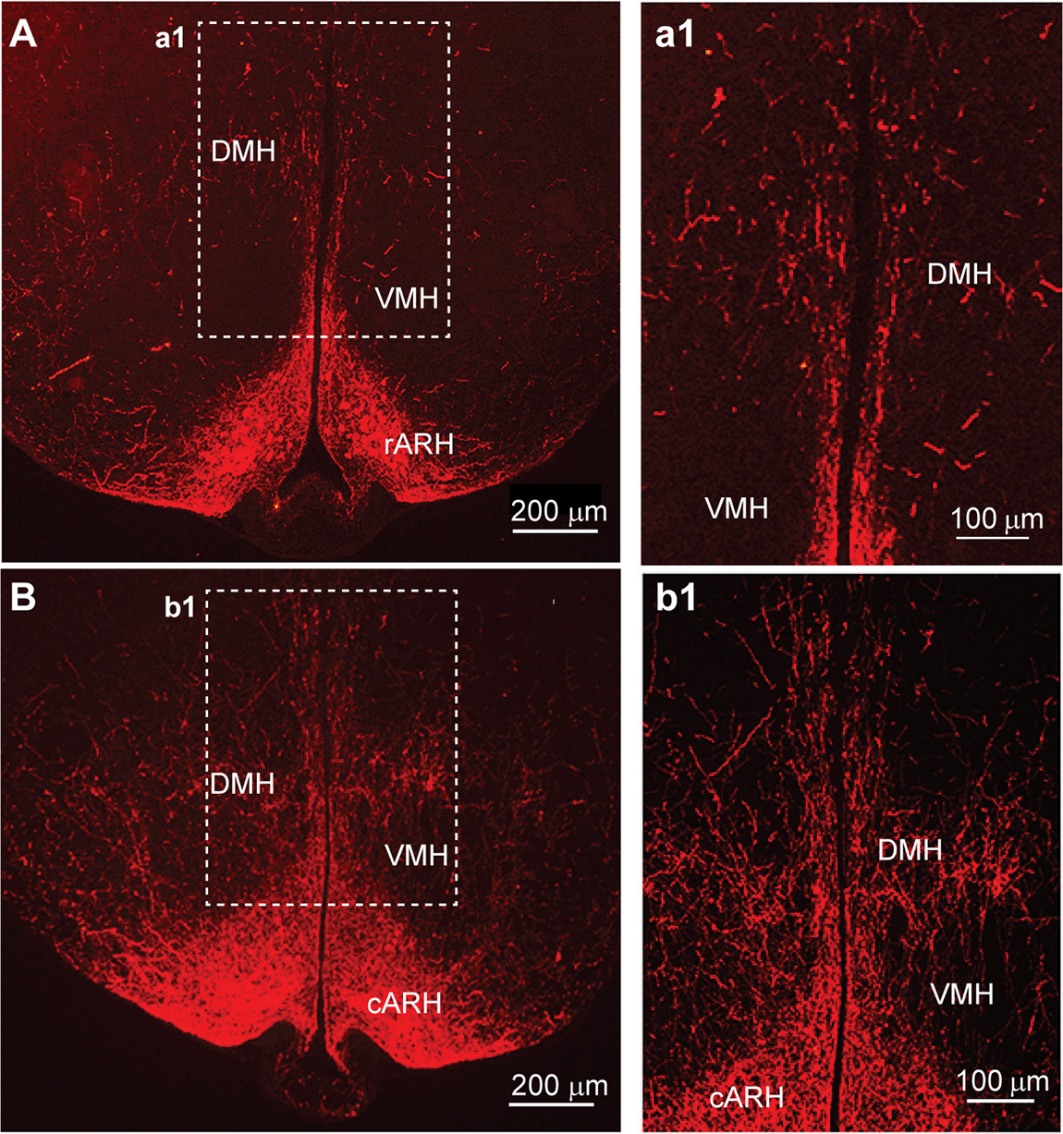
Kiss1^ARH^::ChR2-mCherry projections to the DMH. ***A***, Fluorescent image of ChR2-mCherry fiber-input to the rostral DMH by Kiss1^ARH^ neurons. Inset (***a1***) shows a higher magnification of the stippled area in ***A***. ***B***, Fluorescent image of ChR2-mCherry fiber-input to the caudal DMH by Kiss1^ARH^ neurons. Inset (***b1***) shows a higher magnification of the stippled area in ***B***. DMH, dorsomedial hypothalamic nucleus; LH, lateral hypothalamic area; VMH, ventromedial hypothalamic nucleus; ARH, arcuate hypothalamic nucleus; ME, median eminence. Scale bars: 200 μm (***A***, ***B***) and 100 μm (***a1***, ***b1***).

Unlike Kiss1^AVPV/PeN^ neurons, Kiss1^ARH^ neurons provided only sparse input to the rostral POA where the majority of GnRH neurons are located (Fig. 6*A*,*B*). Importantly, no labeled Kiss1 cell bodies were detected in the AVPV/PeN area (Fig. 6*C*,*D*), and therefore, all projections seen in these sections came from the ARH Kiss1 population. However, Kiss1^ARH^ neurons sent extensive ChR2-mCherry fiber projections to more caudal regions in the POA, primarily to the AVPV, MPO, and the PeN (see also Qiu et al., 2016), and the projections extended dorsolaterally to the BST (Fig. 6*C–E*). In contrast to Kiss1^AVPV/PeN^ projections, Kiss1^ARH^ ChR2-mCherry-labeled fibers were sparse in the VMPO and the MnPO, areas known to be involved in temperature regulation (Fig. 6*A–E*; Tan et al., 2016; Abbott and Saper, 2017). At the level of the rSCN, ChR2-mCherry-labeled fibers from Kiss1^ARH^ neurons appeared to traverse the SCN (Fig. 6*F*), but no direct input to the SCN could be identified at any level. ChR2-mCherry fibers were also traced dorsally to the SPa and into the full extent of the PVH from rostral to caudal (Fig. 7). In addition, labeled fibers from Kiss1^ARH^ neurons were detected in the DMH, particularly in the more caudal regions (Fig. 8). Notably, Kiss1^ARH^ fiber-input to the DMH was considerably less than that from Kiss1^AVPV/PeN^ neurons (Fig. 4 vs Fig. 8). Again, this projection pattern was consistent for all of the Kiss1^ARH^::ChR2-mCherry injected females (*n* = 11).

### Kiss1^AVPV/PeN^ and Kiss1^ARH^ interactions with GnRH neurons and fibers

**Figure 9.**
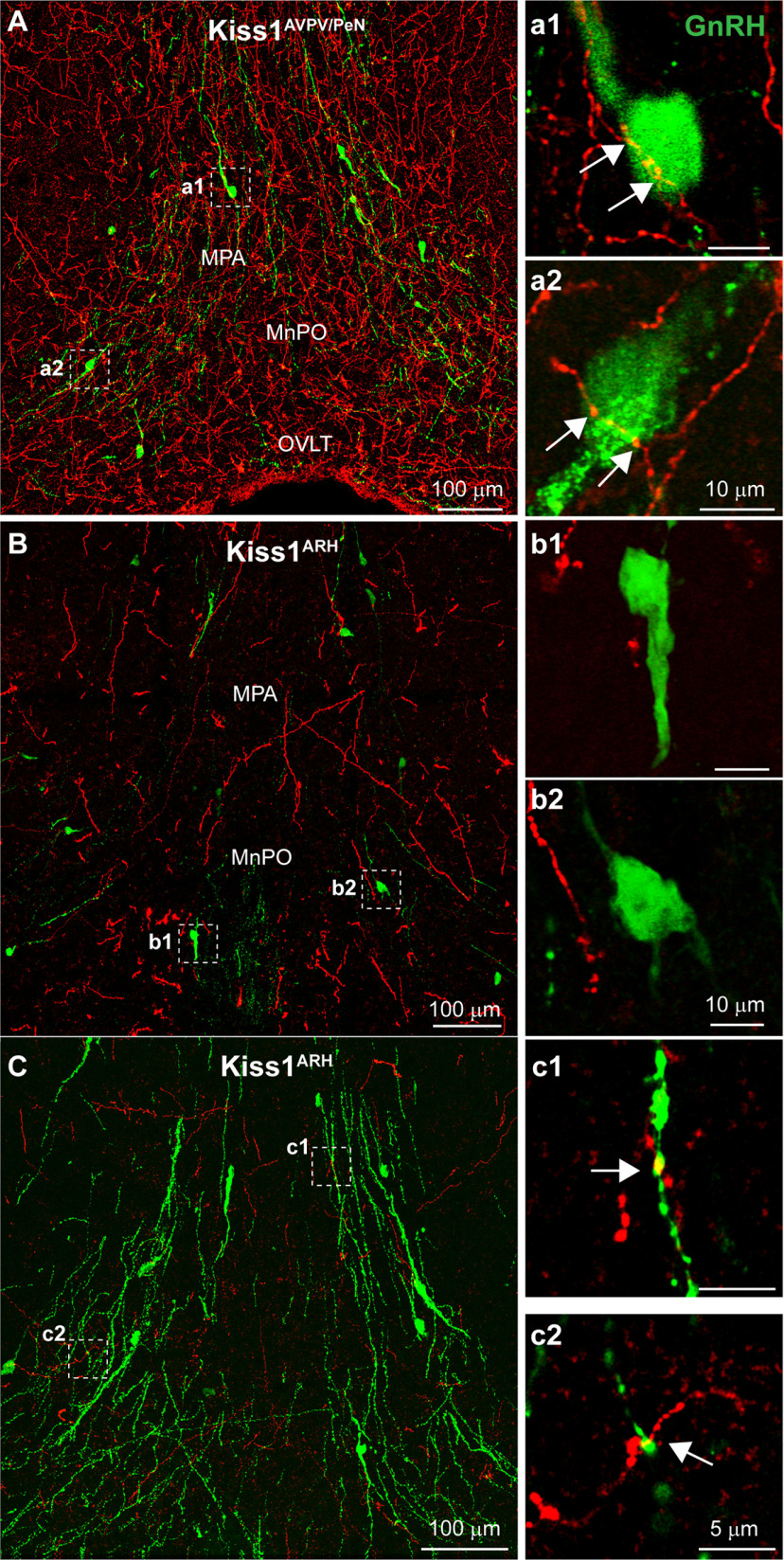
Differential innervation of GnRH neurons by AVPV/PeN and ARH Kiss1 populations. Confocal image montages of immunolabeled ChR2-mCherry Kiss1 fibers and GFP GnRH neurons. ***A***, Kiss1 fibers from the AVPV/PeN are dense in the MPA where GnRH neurons are located. ***a1***, ***a2***, Single optical slice from regions highlighted by white boxes in ***A*** show that GnRH neurons receive close contacts (white arrows) from Kiss1^AVPV/PeN^ fibers. ***B***, Kiss1 fibers from the ARH are scarce in the MPA. ***b1***, ***b2***, Single optical slice (1 μm thick) from regions marked in ***B***. In stark contrast, few Kiss1 fibers from the ARH are present in the MPA or make close apposition with GnRH neurons. ***C***, Confocal image montages of immunolabeled ChR2-mCherry Kiss1 fibers and GnRH neurons identified with the EL14 GnRH antibody, with extensive label of GnRH dendrites (green). ***c1***, ***c2***, Single optical slice from regions highlighted by white boxes in ***C*** show that GnRH neuronal dendrites receive close contacts (white arrows) from Kiss1^ARH^ fibers.

We imaged sections with super-resolution confocal microscopy to search for close contacts or interactions between Kiss1 fibers and GnRH neurons. In order to visualize GnRH neurons and their projections, we co-reacted slides with the EL14 antibody or GFP antibody (see Materials and Methods). Our analysis of GnRH neurons in OVX+E females found that the extensive ChR2-mCherry projections from Kiss1^AVPV/PeN^ neurons (Fig. 9*A*) formed close contacts with the majority (Table 2) of GnRH neurons located within the diagonal band (DB), MS, OVLT/POA area rostrally to the anterior hypothalamus caudally (Fig. 9*a1*,*a2*; Table 2). In contrast, Kiss1^ARH^ neurons provided sparse fiber-input into the rostral brain regions described above (Fig. 9*B*) and onto only a few GnRH neuronal cell bodies (Fig. 9*b1*,*b2*; Table 2). In addition, when using the EL14 (GnRH) antibody to better visualize GnRH processes in addition to soma, there were indications of close contacts between Kiss1^ARH^ fibers and proximal GnRH dendrites (Fig. 9*Cc1*,*c2*).

**Table 2:** Close contact analysis

Injection	Region	Target	Group average	Group SEM	Mice (*n*)	Total CC	Total cells
AVPV	PaLM	OT	33.3%	10.2%	3	34	120
AVPV	PaMM	OT	9.9%	1.4%	3	8	84
AVPV	PV/PaMP	OT	55.6%	8.9%	4	80	126
AVPV	PaLM	AVP	24.2%	3.9%	6	84	342
AVPV	PaMM	AVP	38.1%	7.2%	6	74	287
AVPV	PV/PaMP	AVP	62.2%	6.6%	6	77	125
AVPV	SON	AVP	27.7%	5.0%	3	63	229
AVPV	SON	OT	41.8%	3.8%	3	35	84
AVPV	POA	GnRH	76.7%	3.1%	6	233	327
ARH	PaLM	OT	32.1%	4.1%	4	47	151
ARH	PaMM	OT	25.1%	2.5%	4	42	172
ARH	PV/PaMP	OT	48.3%	5.7%	6	75	146
ARH	PaLM	AVP	33.2%	4.8%	4	86	258
ARH	PaMM	AVP	35.9%	6.7%	5	68	208
ARH	PV/PaMP	AVP	68.6%	9.5%	5	62	88
ARH	POA	GnRH	13.7%	4.5%	4	9	76

Female Kiss1^C^*^re^* or Kiss1^C^*^re^*::GnRH^GFP^ mice were injected with AAV1-ChR2-mCh into either the AVPV/PeN or ARH. Brain sections were processed for double-label ICC to enhance the mCherry signal and visualize potential postsynaptic targets of Kiss1 fibers using confocal microscopy. The PVH was divided into the dorsolateral PaLM, ventromedial PaMM, and the medial PV/PaMP regions. GnRH neurons in the rostral forebrain (POA), comprising the DB, MS, POA, and anterior hypothalamus were analyzed. Additionally, the supraoptic nucleus (SON) was also examined. The postsynaptic populations included OT, AVP, and GnRH neurons.

**Table 3:** Key resources table

Reagent type (species) or resource additional information		Designation	Source or reference	Identifiers
Genetic reagent (*M. Musculus*)	Kiss1^C^*^re^*^:GFP^ version 2 (V2)	Dr. Richard D. Palmiter; University of Washington; PMID: 29336844		
Genetic reagent (*M. Musculus*)	*GnRH ^GFP^*	Suter et al. (2000); PMID:10614664		
Genetic reagent (AAV)	AAV1-Ef1α-DIO-ChR2:mCherry	Dr. Stephanie L. Padilla; University of Washington; PMID: 25429312		
Antibody	Anti-mCherry (rabbit polyclonal )	Abcam	Abcam: ab167453 RRID:AB_2571870	(1:10 000)
Antibody	Anti-mCherry (goat polyclonal)	Biorbyt	Biorbyt: orb11618 RRID:AB_2687829	(1:10 000)
Antibody	Anti-kisspeptin (rabbit polyclonal)	Dr. Alain Caraty Universite Francois- Rabelais Tours; PMID:16621281	No. 564 RRID: AB_2622231	(1:2500)
Antibody	Anti-OT (rabbit polyclonal)	Morris et al. (1980); PMID:7448278		(1:5000)
Antibody	Anti-GnRH (rabbit polyclonal)	Ellinwood et al. (1985); PMID:3887335	EL-14 RRID:AB_2715535	(1:5000)
Antibody	Anti-AVP (rabbit polyclonal)	Dave et al. (1985); PMID: 2992912		(1:5000)
Antibody	Biotin-SP AffiniPure bovine anti-goat IgG	Jackson ImmunoResearch Labs	Jackson: 805-065-180 RRID: AB_2340876	(1:500)
Antibody	Biotin anti-GFP (goat polyclonal)	Abcam	Abcam: ab6658 RRID: AB_305631	(1:5000)
Antibody	Alexa Fluor 594-streptavidin	Jackson ImmunoResearch Labs	Jackson: 016-580-084 RRID: AB_2337250	(1:2500)
Antibody	Goat anti-rabbit IgG Alexa Fluor 594	Thermo Fisher Scientific	Catalog #A-11037 RRID: AB_2534095	(1:500)
Antibody	Goat anti-rabbit IgG Alexa Fluor 488	Thermo Fisher Scientific	Catalog #A-11034 RRID: AB_2576217	(1:1000)
Software	ZEN digital imaging for light microscopy	Zeiss	RRID:SCR_013672	

**Figure 10.**
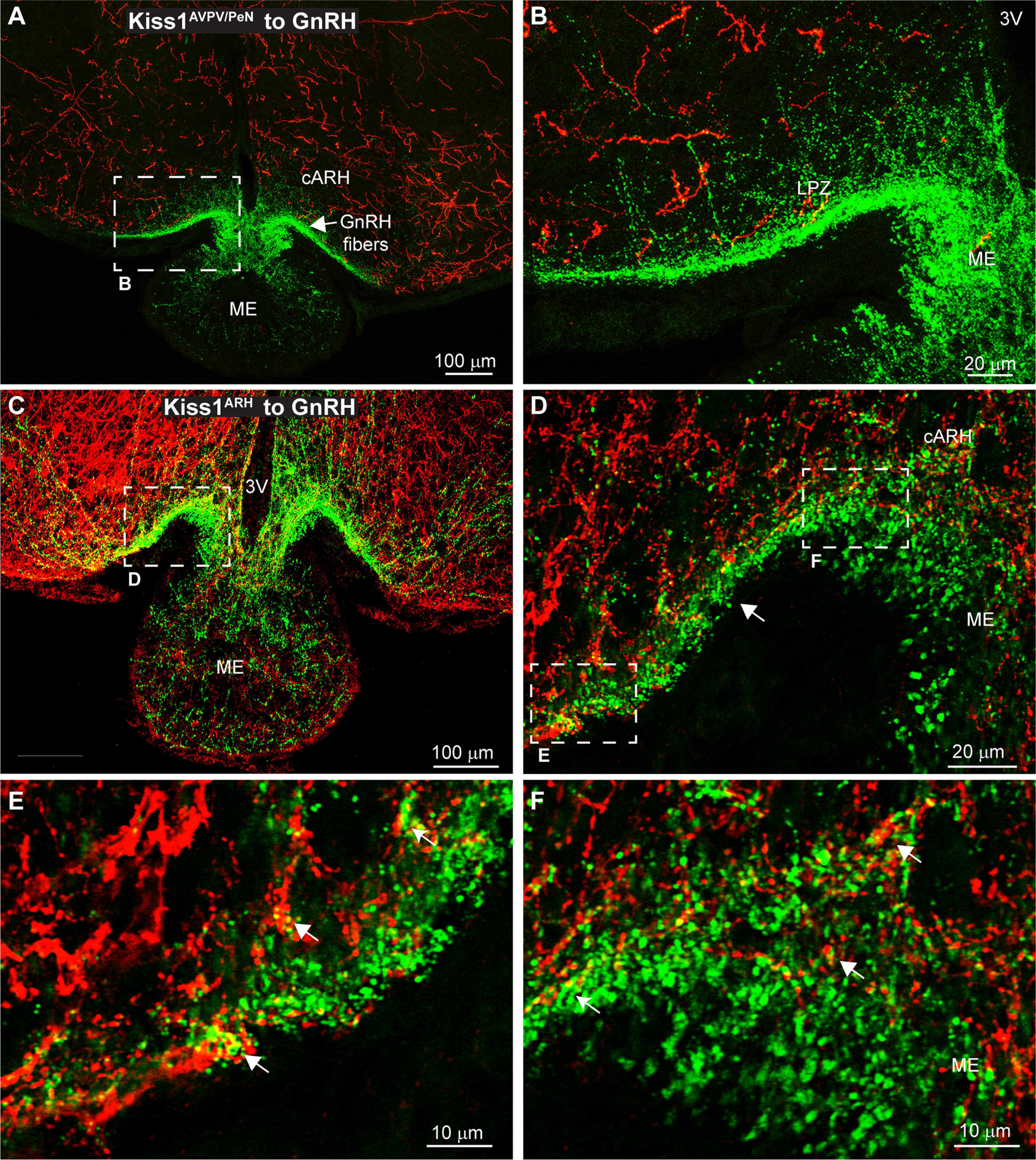
Interactions between GnRH and Kiss1 fibers in the ME. Confocal image montages of double label ICC that show Kiss1 fibers (ChR2-mCherry) originating from either the AVPV/PeN or ARH. GnRH fibers (green) enter the ARH laterally and along the 3V from the POA before entering the ME. ***A***, Kiss1^AVPV/Pen^ fibers are diffuse in the ARH and do not enter the ME. ***B***, Expanded view of the dashed box from ***A***. Few Kiss1^AVPV/PeN^ fibers reach the ventral surface where GnRH fibers concentrate, which would suggest interactions are unlikely. ***C***, Confocal image montage displays staining of Kiss1^ARH^ cell bodies and projections in the cARH and ME. ***D***, Single optical slice (1 μm thick) of the region outlined in ***C***. GnRH fibers enter the ARH laterally (left) to briefly form a bundle (white arrow) that runs along the ventral surface before dispersing in the ME. ***D***, Expanded view of the left white box in ***C***. The occasional presence of yellow pixels and lack of black pixels between Kiss1^ARH^ and GnRH fibers suggests the presence of interactions. ***E***, ***F***, Expanded view of the regions from the white boxes in ***D***. As the GnRH fibers enter the ME the lack of black pixels between Kiss1^ARH^::ChR2-mCherry and GnRH fibers and presence of yellow pixels suggest close contacts (white arrows) between fibers.

Kiss1^AVPV/PeN^ neurons provided negligible input to the ME (Figs. 4, 10*A*), and as such, few Kiss1^AVPV/PeN^ fibers were seen in close proximity to GnRH fibers in this region (Fig. 10*B*). Clearly a primary target of the Kiss1^AVPV/PeN^ neurons are the GnRH neuronal somas (Fig. 9*A*) and not the fiber-terminals in the ME (Fig. 10*A*,*B*). Conversely, Kiss1^ARH^ neurons project throughout the ME, including the external layer, although the major projections were directly onto the lateral palisade zone (LPZ) where GnRH fibers are highly concentrated (Figs. 5, 10*C–F*; King et al., 1982; Rønnekleiv and Kelly, 1986). The maximum intensity of the projection (Fig. 10*C*) gives the impression of a significant interaction between Kiss1^ARH^ and GnRH fibers; however, when viewed in a single confocal plane (Fig. 10*D*), the ChR2-mCherry and GnRH fibers mostly appeared to run in parallel (white arrow). Still, yellow pixels designating close contacts of mCherry and GFP could be seen laterally where the GnRH fibers consolidate (Fig. 10*E*) and also immediately before entering the ME (Fig. 10*F*). Thus, the occasional close contacts between Kiss1^ARH^ and GnRH fibers were primarily observed at the lateral interface of the ARH with the stalk ME and most likely represent non-synaptic points of close interaction/proximity between Kiss1^ARH^ and GnRH fibers (Uenoyama et al., 2011; Liu et al., 2021).

### Kiss1^AVPV/PeN^::ChR2-mCherry-labeled fibers primarily onto OT neurons in the SON

**Figure 11.**
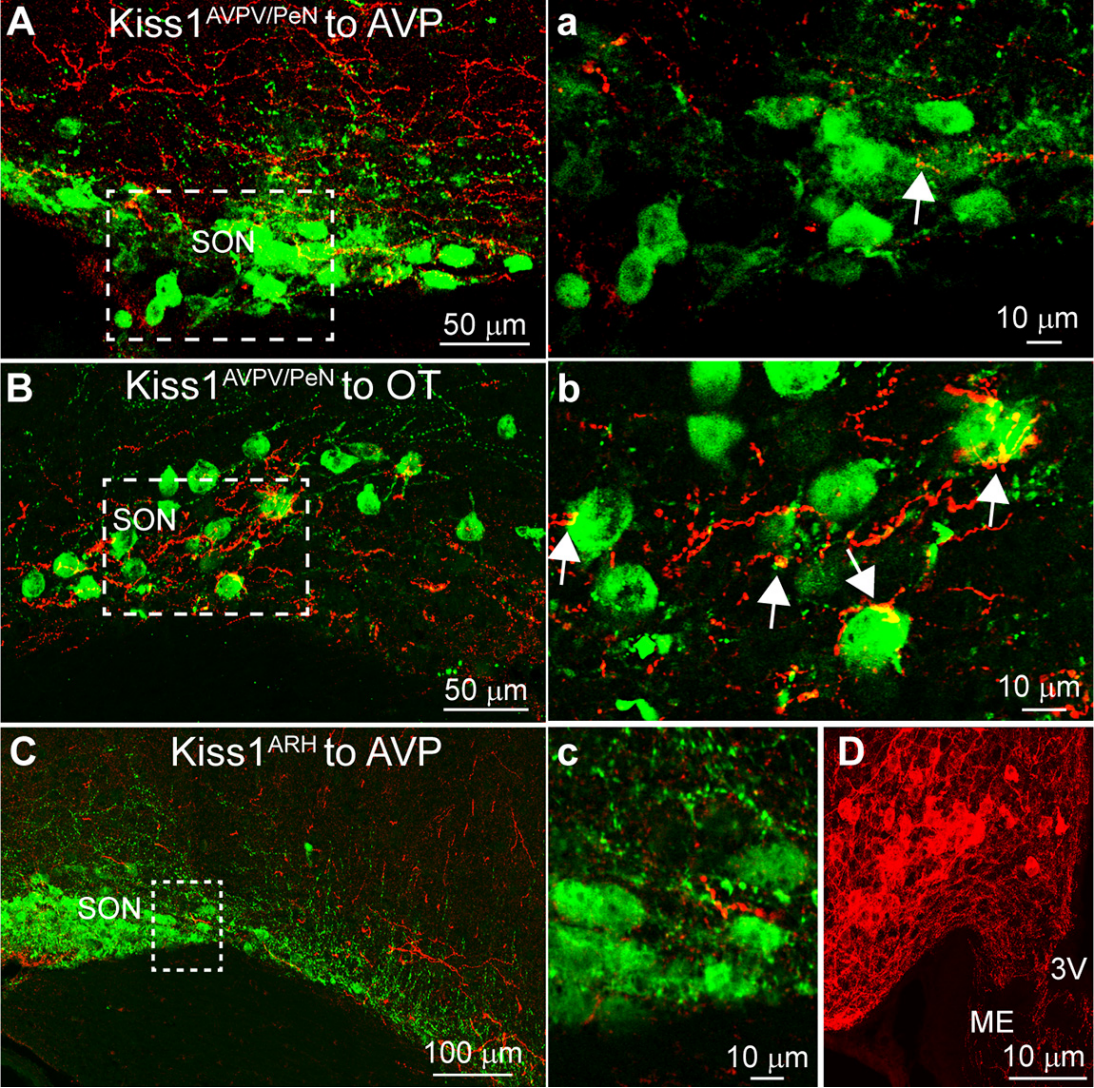
Kiss1 fiber projections to AVP and OT neurons in the SON. ***A***, Confocal image montage of double-label ICC that shows Kiss1^AVPV/PeN^ fibers (ChR2-mCherry) run laterally along the ventral surface to reach the SON. A population of AVP neurons (green) are found just above and lateral to the optic tract in the SON. ***a***, Single optical slice of view from region marked by a white box in ***A***. Despite many nearby Kiss1^AVPV/PeN^ fibers there are few indications of close contacts. ***B***, Slightly medial to the AVP neurons, a population of OT neurons is also located in the SON. ***b***, Single optical slice analysis at 1-μm focal plane shows that Kiss1^AVPV/PeN^ fibers are more likely to make contact with OT neurons and appositions are quite extensive (white arrows). ***C***, Kiss1^ARH^ fibers do not provide notable innervation of the SON. ***c***, Single confocal plane of region marked in white box. Few if any SON AVP neurons receive close contacts. ***D***, Confocal image of mCherry-labeled Kiss1 neurons in the ARH from the same animal as in ***C***. The lack of Kiss1^ARH^ fibers to the SON was seen even when Kiss1^ARH^ neurons were strongly labeled (***D***).

ChR2-mCherry-labeled fibers from Kiss1^AVPV/PeN^ neurons were found to extend laterally toward the medial parts of the SON (Fig. 2*D*). As both AVP and OT neurons are found in the SON, we used confocal microscopy in conjunction with double-label ICC to determine whether Kiss1 fibers made contact with either population. Super-resolution images confirmed that fibers did reach and were present in the SON (Fig. 11*Aa*,*Bb*). Through analysis of the confocal Z-stack we were also able to identify close contacts made onto SON neurons. Specifically, yellow pixels, where Kiss1 fibers met with cell bodies, were seen on roughly one-third of AVP neurons (Table 2), which occurred more frequently with OT neurons (Table 2). However, the close contacts that were seen onto VP neurons appeared more rarely (Fig. 11*Aa*), whereas those onto OT neurons were more extensive (Fig. 11*Bb*). Therefore, the number of contacts with AVP neurons is likely an over estimation, but we are more confident that Kiss1^AVPV/PeN^ fibers make close contact with OT neurons. We did attempt to examine close contacts between Kiss1^ARH^ fibers and OT/VP neurons; however, these projections barely reached the SON (Fig. 11*C*). Even the more medial AVP neurons, relative to the location of OT SON neurons, lacked significant contacts (Fig. 11*Cc*). The absence of fibers in the SON was seen even with “on target” ARH injections and strong ChR2-mCherry expression (Fig. 11*D*). Therefore, we did not proceed further with this particular analysis.

### Kiss1^AVPV/PeN^ and Kiss1^ARH^ projections to AVP neurons in the SCN

**Figure 12.**
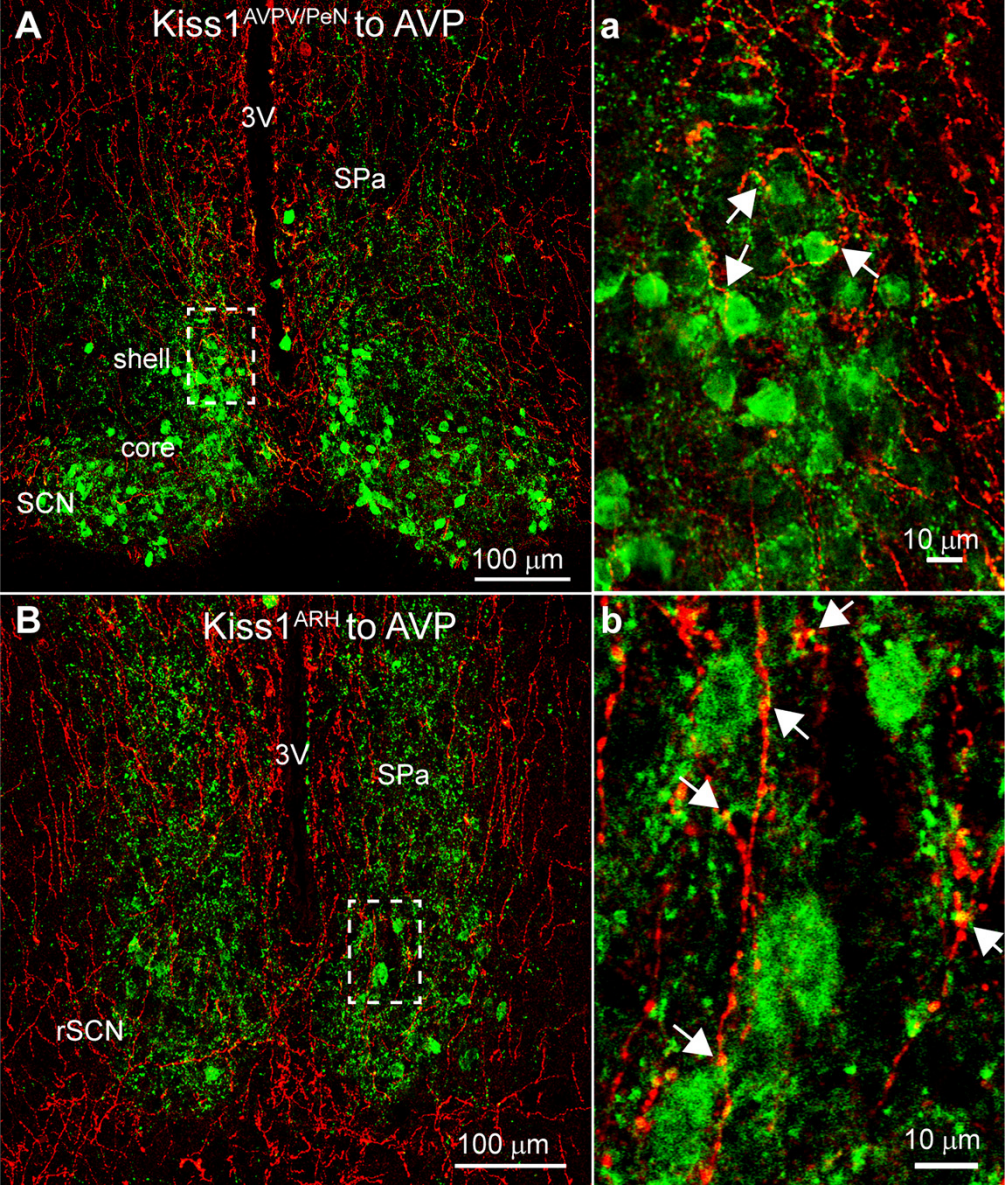
Kiss1 fiber projections to AVP neurons in the SCN. *A*) Confocal image montage shows that Kiss1^AVPV/PeN^ fibers (red) run along the 3V and surround the SCN. Double label ICC was used to visualize AVP neurons (green) which are primarily located in the shell of the SCN. ***a***, Although practically no Kiss1^AVPV/PeN^ fibers enter the SCN core, when viewing a single optical plane close contacts appear to be made with some of the most external AVP neurons (white arrows). ***B***, Confocal image montage of the Kiss1^ARH^ fibers in the rSCN. Few AVP cells bodies are present, but ascending projections can be seen running parallel to the 3V. ***b***, Single optical slice centered on the region demarcated by the white box in ***B***. A number of Kiss1^ARH^ fibers pass through the SCN, potentially making close contact with AVP neurons or their fiber projections. However, the density of labeled AVP projections obscures somata and hinders analysis.

While OT neurons are not present in the SCN, AVP neurons are known to reside in the SCN, particularly the shell region (Fig. 12*A*). Kiss1^AVPV/PeN^::ChR2-mCherry-labeled fibers largely avoided the core of the SCN (Figs. 2*D*, 3*A*), but appeared to contact the most dorso-medial AVP cells (Fig. 12*a*). Similarly, Kiss1^ARH^::ChR2-mCherry-labeled fibers also tended to avoid the SCN, but a subset of fibers could be seen passing through the rostral SCN (Fig. 12*B*). Examination of single confocal planes did reveal a few potential close contacts (Fig. 12*b*), but the large number of local AVP processes made it difficult to distinguish between Kiss1 fibers passing through the SCN and those actually making close contact.

### Kiss1 input onto OT neurons in the PVH

**Figure 13.**
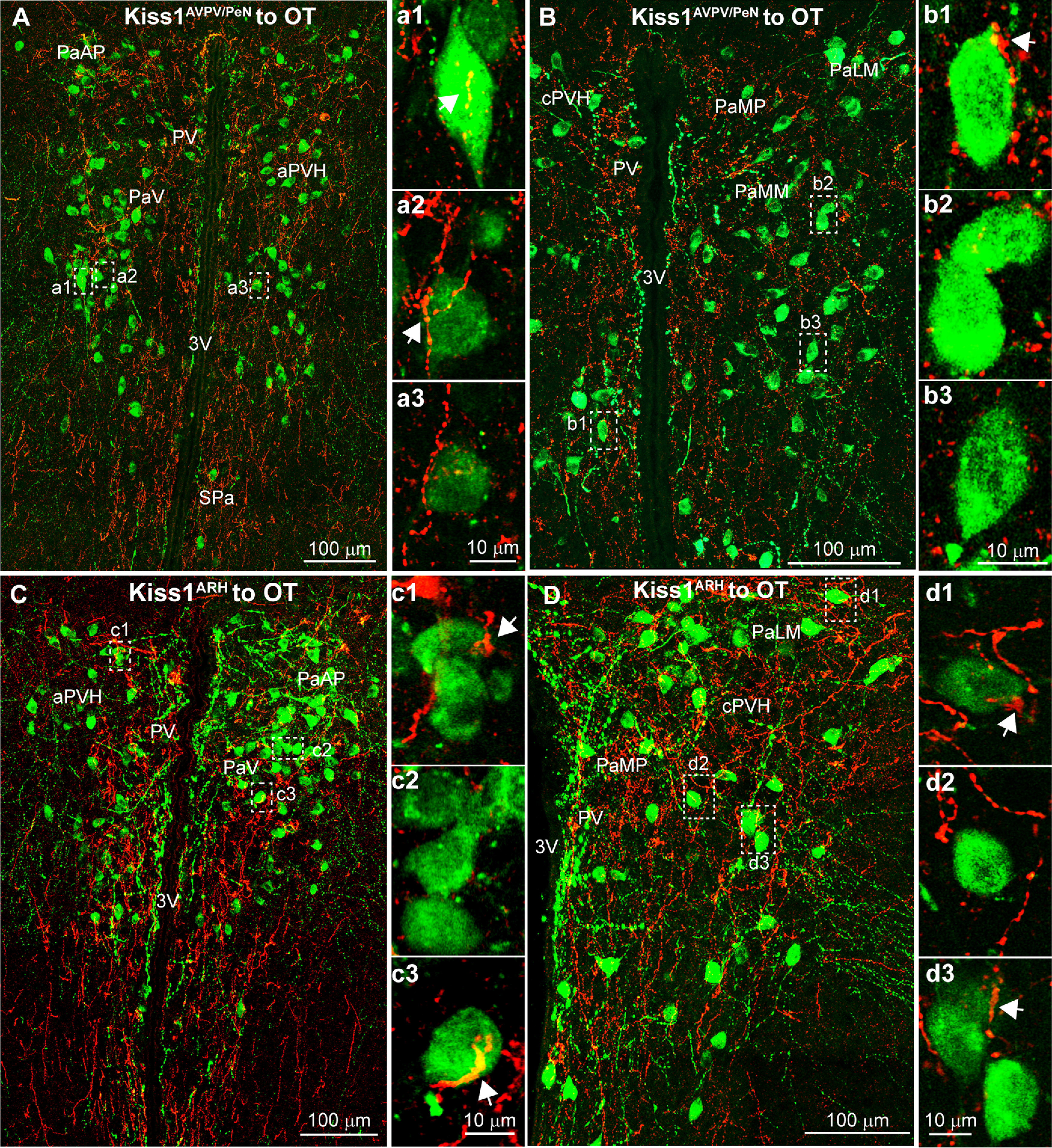
Kiss1 fiber projections to OT neurons in the PVH. Confocal image montage of Kiss1^Cre^::ChR2-mCherry fibers in the PVH. Double label ICC was used to visualize mCherry (red) and OT neurons (green). ***A***, ***C***, In the aPVH, OT neurons are primarily located in the PaAP and the PaV regions with a smaller number present in the in the PV. ***a1–a3***, Focused views of a single optical slice of individual neurons from ***A***. ***c1–c3***, Focused views of a single optical slice of individual neurons from ***C***. ***B***, ***D***, In the cPVH, OT neurons are diffusely distributed through most the PVH. ***b1–b3***, Focused views of single optical slice ***B***. ***d1–d3***, Images from single optical slice in ***D***. See Table 2 for summary of the close contact analysis. For abbreviations, see Figures 3, 7.

OT neurons are located in different subdivisions of the PVH, including the paraventricular medial parvicellular (PaMP) regions, as well as dorsolateral (PaLM) and ventromedial magnocellular (PaMM) parts, as previously described (Biag et al., 2012). In the aPVH, the OT neurons are scattered throughout the nucleus with a small number of cells found near the third ventricle (Fig. 13*A*,*C*). Using super-resolution confocal microscopy, we assessed the presence of close contacts made between ChR2-mCherry labeled Kiss1 fibers from either the AVPV/PeN (Fig. 13*A*,*a1–a3*) or the ARH (Fig. 13*C*,*c1–c3*). We found that Kiss1^AVPV/PeN^ fibers made close appositions with an average of 42.1% (99/235 cells; *n* = 2) and Kiss1^ARH^ with 25.1% (58/231 cells; *n* = 3) of the OT neurons in the aPVH. Further caudal, the ChR2-mCherry fiber-input to the PVH thins and spreads laterally into “wings” of the PVH (Figs. 3, 7). Here, in the cPVH, Kiss1^AVPV/PeN^ mCherry fibers were primarily found along the ventricle with fewer fibers extending laterally (Fig. 13*B*) and, therefore, the periventricular/medial population of OT neurons (PV/PaMP; Table 2) received more close contacts than the dorsolateral PaLM (Table 2) and ventromedial PaMM (Table 2). In comparison, Kiss1^ARH^ fibers were more evenly distributed throughout the cPVH (Fig. 13*D*) and show a similar likelihood of contacting either the PV/PaMP (Table 2), PaLM (Table 2), or PaMM (Table 2) OT neurons.

### Kiss1 input onto AVP neurons in the PVH

**Figure 14.**
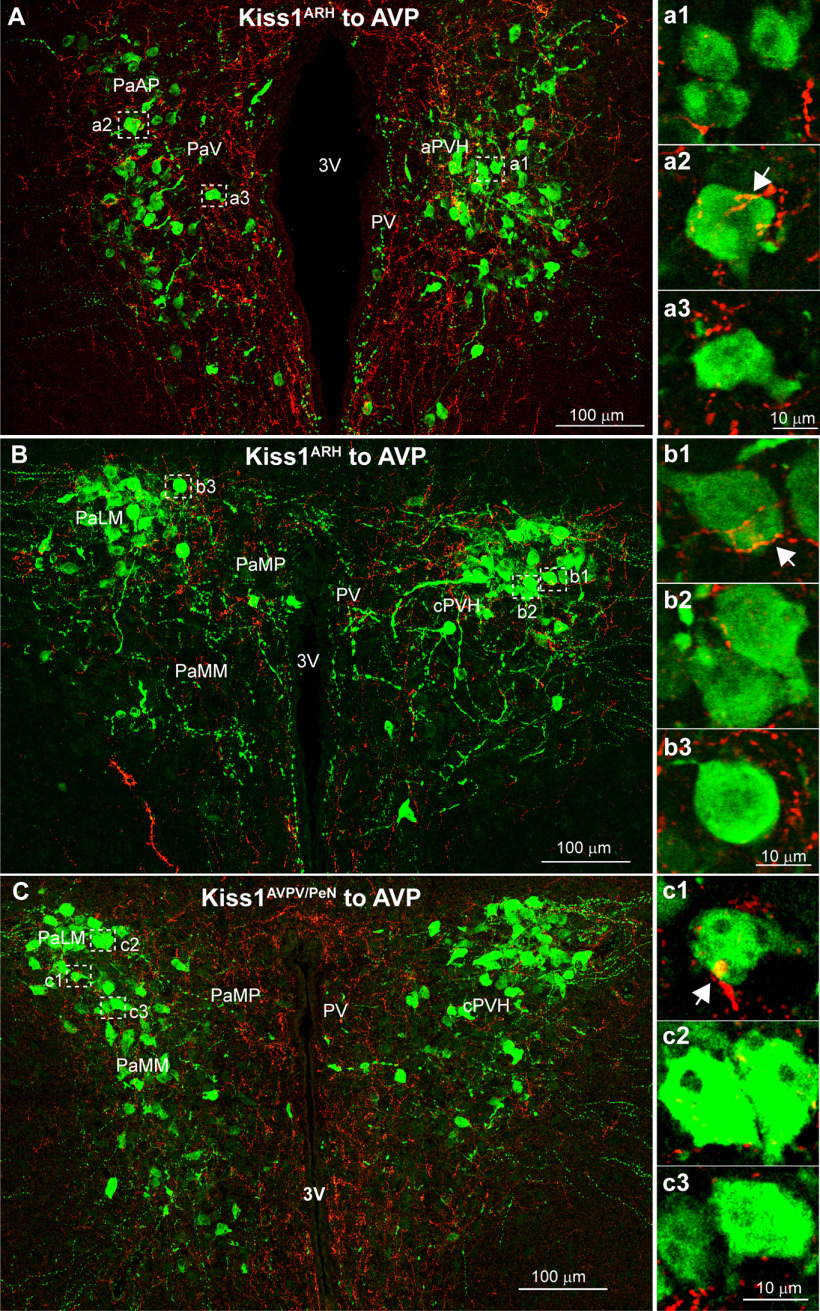
Kiss1 fiber projections to AVP neurons in the PVH. AVP neurons are also present in the aPVH but tend to be found more laterally with fewer cells near the 3V. ***A*,** AVP neurons are located in parvicellular regions of the aPVH such as the PaAP and PaV. ***a1–a3***, Single optical plane images of Kiss1^ARH^::ChR2-mCherry fibers that make close contact with AVP neurons. ***B***, In the more caudal PVH, AVP neurons are confined to tight clusters of cells in the PaLM. ***b1–b3*,** Single optical plane images reveal that Kiss1^ARH^ fibers make close contact with AVP neurons in the PaLM. ***C***, Kiss1^AVPV/PeN^ input onto AVP neurons were only investigated in the cPVH. A confocal image montage shows that many AVP neurons (green) are clustered in the lateral regions where Kiss1^AVPV/PeN^ fibers make contact with AVP neurons in the PaLM and PaMM magnocellular regions. ***c1–c3***, Single optical plane images focused on individual AVP cells from ***C*** demarcated by white boxes. See Table 2 for summary of the close contact analysis.

Similar to OT neurons, AVP neurons were also observed in the periventricular (PV) area dorsal to the SCN, the PaAp area in the aPVH and further caudal in different subdivisions of the cPVH, including the PaMP parvicellular regions, as well as dorsolateral (PaLM) and ventromedial (PaMM) magnocellular parts. However, AVP neurons tended to be found more laterally, with only a few neurons located near the ventricle (Fig. 14). Kiss1^ARH^ fibers made close contact with roughly a third of PaLM (Table 2) and PaMM (Table 2) AVP neurons. Despite the smaller AVP population in PV/PaMP, the majority there received close contacts from Kiss1^ARH^ neurons (Table 2). Kiss1^AVPV/PeN^ fibers into the PVH (Fig. 14*C*) primarily contacted the PV/PaMP (Table 2) with the PaMM (Table 2) and PaLM (Table 2) receiving fewer close contacts. Clearly, ChR2-mCherry Kiss1^AVPV/PeN^ and Kiss1^ARH^ fibers may contact other neurons besides those expressing OT and AVP within the PVH, such as those expressing prodynorphin (pDyn), corticotropin-releasing factor (CRF), melanocortin-4 receptor (MC4R), and/or vGluT2, based on the distribution of these neurons within the PVH as shown using animals expressing the respective Cre recombinase (Viau and Sawchenko, 2002; Xu et al., 2013; Garfield et al., 2015). Indeed, some of these were identified using scRT-PCR of harvested neurons following whole-cell recording.

### Kiss1^AVPV/PeN^ neurons inhibit PVH and DMH neurons via GABA release

**Figure 15.**
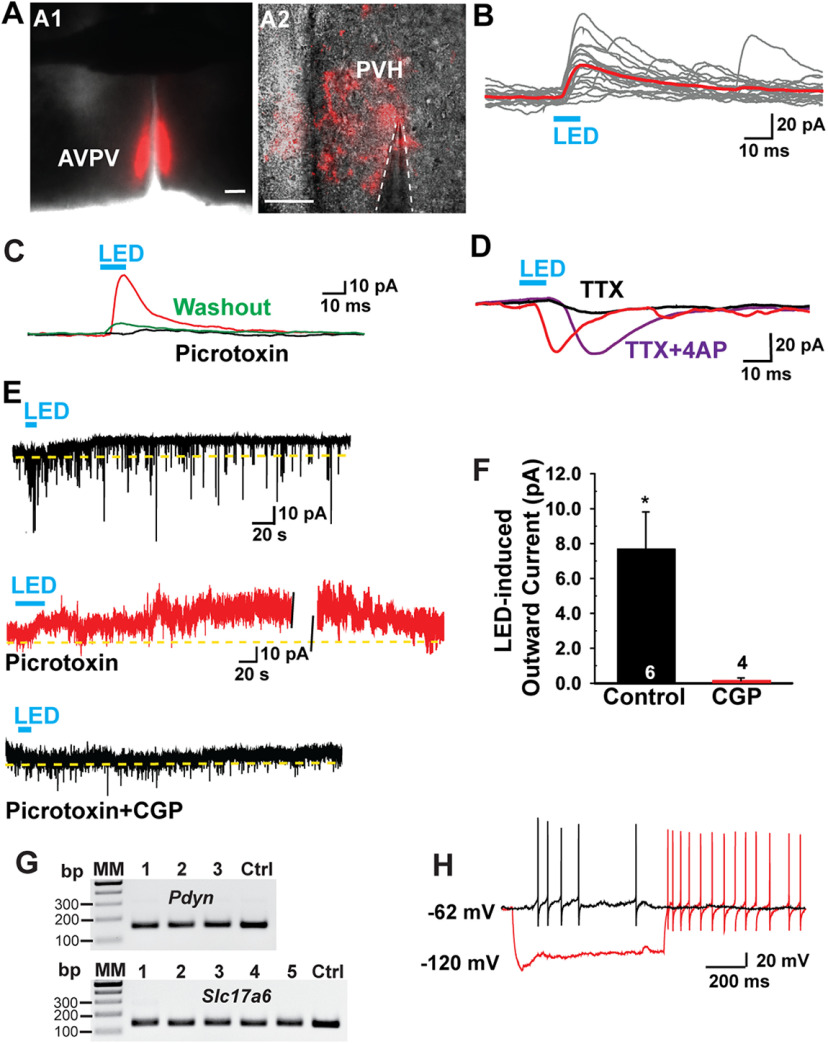
Postsynaptic responses to optogenetic stimulation of Kiss1 ^AVPV/PeN^ fibers release GABA onto PVH neurons. ***A1***, Low-power composite image of a coronal slice through AVPV. Injections of AAV-DIO:ChR2:mCherry into the AVPV/PeN labeled Kiss1^AVPV/PeN^ neurons from rostral to caudal. ***A2***, High-power composite image shows fluorescence projections are visible around the patched cells in the PVH. Low-power scale bar: 200 μm (***A1***); high-power scale bar: 40 μm (***A2***). ***B–C***, Using a standard internal solution, whole-cell voltage clamp recordings were made in PVH neurons (V_hold_ = −10 mV). ***B***, Fast outward currents were seen in response to blue light stimulation. Red trace is the averaged response. ***C***, The GABA_A_ antagonist picrotoxin (100 μm) effectively and reversibly blocked the optogenetically evoked outward currents to confirm GABAergic signaling. ***D***, The optogenetically evoked currents were also blocked by TTX (1 μm), but recovered when the K^+^ blockers 4-AP (0.5 mm) and TEA (7.5 mm) were added to the bath when using high chloride internal solution (Vhold = −60 mV). This “rescue” of the response is physiological evidence of a direct synaptic connection. ***E***, High-frequency optogenetic stimulation (20 Hz, 10 s) generated a slow IPSC in a PVH neuron using high chloride internal solution (V_hold_ = −60 mV; upper trace). In another cell, after blocking the LED-induced GABA_A_ responses with picrotoxin at 100 μm, high-frequency optogenetic stimulation (20 Hz, 30 s) using normal internal solution still generated a slow IPSC that recovered in 10 min (middle trace). Furthermore, the GABA_B_ antagonist CGP 55845 (1 μm) blocked the slow IPSC response in the presence of picrotoxin in another cell (bottom trace). Blue bar below or above the recordings indicate LED stimulus (***B–E***). ***F***, Summary of the effects of CGP 55845 on the high-frequency optogenetic stimulation (20 Hz, 10 s)-induced slow IPSC in PVH neurons (un-paired *t* test, *t*_(8)_ = 2.826, *p = 0.0223). Data points represent the mean ± SEM. Cell numbers are indicated. ***G***, Representative gel images of scRT-PCR *Pdyn* and *Vglut2* mRNA expression in eight responsive PVH neurons. MM, molecular markers; 1–3 and 1–5, the recorded PVH neurons; Ctrl, positive tissue control. ***H***, A representative trace of a Pdyn neuron showing a “sag” (h-current) with hyperpolarizing current injection and rebound burst firing in current clamp. Two of three *Pdyn* neurons exhibited an h-current.

**Figure 16.**
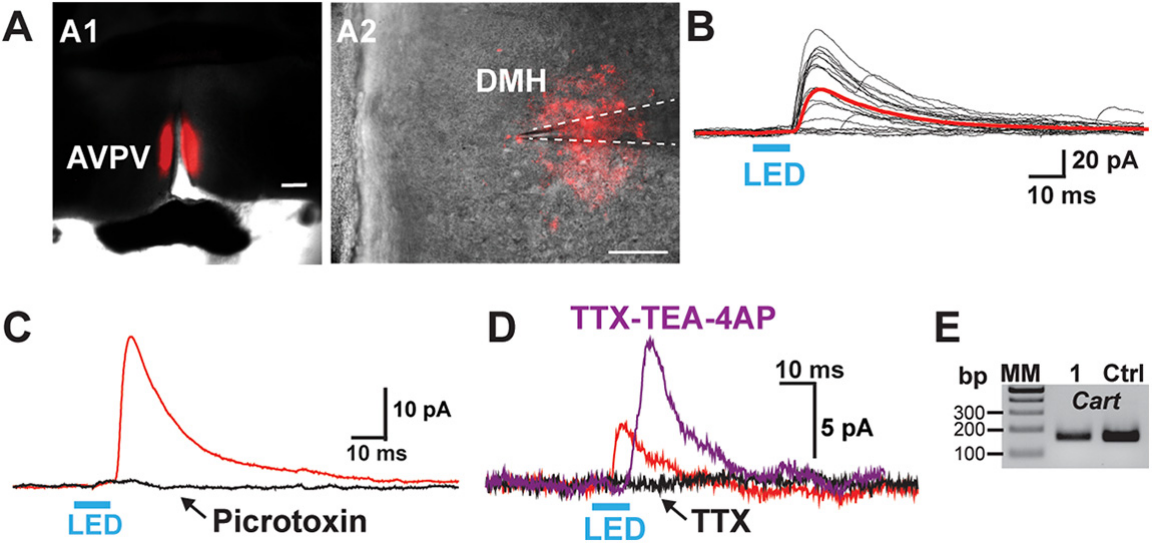
Postsynaptic responses to optogenetic stimulation of Kiss1 ^AVPV/PeN^ fibers release GABA onto DMH neurons. ***A1***, Low-power composite image of a coronal slice through AVPV. Injections of AAV-DIO:ChR2:mCherry into the AVPV/PeN labeled Kiss1^AVPV/PeN^ neurons from rostral to caudal. High-power composite image shows fluorescence projections are visible around the patched cells in the DMH (***A2***). Low-power scale bar: 200 μm (***A1***); high-power scale bar: 40 μm (***A2***). ***B–D***, Using a standard internal solution, whole-cell voltage clamp recordings were made in DMH neurons (V_hold_ = −10 mV). ***B***, Fast outward currents were seen in response to blue light stimulation. Red trace is the averaged response. ***C***, The outward currents were blocked by picrotoxin (100 μm), demonstrating that currents were GABAergic. ***D***, Baseline postsynaptic responses (red) were blocked by TTX (black), but rescued after K^+^ blockers 4-AP and TEA (magenta) were added to the bath, indicating a direct synaptic connection between the Kiss1^AVPV/PeN^ and DMH neurons. Blue bar below the recordings indicates LED stimulus (***B–D***). ***E***, Gel image of *Cart* mRNA expression of the “rescued” DMH neuron in ***D***. MM, molecular markers; 1, the recorded DMH cell; Ctrl, positive tissue control.

As a complementary approach, we did whole-cell, voltage-clamp recordings in slices from E2-treated OVX female mice, and examined evoked (photostimulated) postsynaptic currents (PSCs) in PVH and DMH neurons from Kiss1^Cre:GFP^ mice that had received bilateral injections of AAV1-ChR2-mCherry into AVPV/PeN (Figs. 15, 16*A1*,*A2*). It had been shown previously that Kiss1^AVPV/PeN^ neurons express *Slc32a1* (vGAT) and not *Slc17a6* (vGluT2) mRNA in the animal model used for these experiments (Qiu et al., 2016). To confirm these data, we harvested *in vitro* 30 Kiss1^AVPV/PeN^ neurons from each of two OVX, E2-treated Kiss1^CreGFP^ females. The scRT-PCR analysis revealed that 70–80% of the harvested Kiss1 neurons expressed *Slc32a1* mRNA and only 3.3% (two of 60) were found to express *Slc17a6*. Therefore, these data confirm that GABA and not glutamate is packaged into vesicles and released by Kiss1^AVPV/PeN^ neurons in this animal model (Qiu et al., 2016).

Using a standard internal solution, whole-cell voltage clamp recordings were made in PVH neurons (V_hold_ = −10 mV). Fast outward currents were recorded in response to blue light stimulation in 23 PVH cells (Fig. 15*B*). This light-induced response was abrogated (91% blockade) with picrotoxin (100 μm), a GABA_A_ receptor antagonist (Fig. 15*C*). To augment the GABA-mediated response from Kiss1^AVPV/PeN^ neurons, we recorded an additional cohort of neurons using a high chloride internal solution (*n* = 10), which shifted E_Cl–_ and changed the GABA-mediated response from an outward to an inward current (Fig. 15*D*,*E*), which was also blocked by picrotoxin (12.4 ± 0.4 vs 140.0 ± 34.5 pA control, *n* = 3). The small latency between optogenetic stimulation and postsynaptic response (4.5 ± 0.3 ms, *n* = 31) was suggestive of a direct connection (Felix-Ortiz et al., 2013; Holloway et al., 2013). However, to further support this supposition we blocked the photostimulated postsynaptic inward current with TTX (Fig. 15*D*) and rescued the light-induced response with the addition of K^+^ channel blockers 4-AP and TEA (Fig. 15*D*; Cousin and Robinson, 2000; Petreanu et al., 2009), which is biophysical evidence for direct synaptic contact between Kiss1^AVPV/PeN^ neurons and PVH parvocellular neurons. With the rescue experiment there was a twofold increase in the latency (*n* = 4) to the response because of the fact that the calcium influx into the nerve terminal is principally dependent on ChR2 when the fast sodium channels are blocked by TTX (Felix-Ortiz et al., 2013; Holloway et al., 2013).

Furthermore, in voltage clamp (V_hold_ = −60 mV) high-frequency stimulation of Kiss1^Cre^-ChR2 fibers generated a slow IPSC in PVH neurons (Fig. 15*E*), which was blocked by GABA_B_ receptor antagonist CGP 55845 (1 μm; Fig. 15*F*). The PSC responsive neurons were located dorso-medially in the PVH with a resting membrane potential of −64.9 ± 2.3 mV, an input resistance of 1.2 ± 0.1 GΩ and a capacitance of 20.4 ± 1.0 pF (*n* = 29), typical endogenous properties of parvocellular neurons (Luther and Tasker, 2000; Stern, 2001). Indeed, ten neurons were identified *post hoc* via scRT-PCR as expressing *Vglut2* mRNA, and four neurons expressed *Pdyn* mRNA, two of which also expressed *Vglut2* (Fig. 15*G*) The *Pdyn* PVH neurons had an input resistance of 1.6 ± 0.4 GΩ and cell capacitance of 16.8 ± 2.2 pF. The majority (70%) of the responsive PVH neurons, expressed an h-current, including the *Pdyn* neurons (Fig. 15*H*), which is an endogenous characteristic of preautonomic parvocellular PVH neurons (Stern, 2001).

Kiss1^AVPV/PeN^ neurons also inhibited DMH neurons via GABA release. Blind whole-cell patch was done on DMH neurons near Kiss1-ChR2:mCherry fibers (Fig. 16*A2***)** in OVX+E2 mice that received bilateral injections of AAV1-ChR2-mCherry into the AVPV/PeN area (Fig. 16*A1*). Outward PSCs were reliably evoked by photostimulation and sufficiently strong enough to be measured using a standard K^+^ gluconate internal solution (V_hold_ = –10 mV) in 24 DMH neurons (Fig. 16*B–D*). Picrotoxin was effective at blocking the currents, confirming GABAergic signaling (Fig. 16*C*). The parvocellular neurons displaying an inhibitory PSC had an average resting membrane potential of −62.6 ± 2.3 mV, input resistance of 1.5 ± 0.2 GΩ, and cell capacitance of 14.1 ± 1.0 pF (*n* = 10). Similar to the GABAergic Kiss1^AVPV/PeN^ inputs to PVH, the latency to response onset in DMH cells was 4.5 ± 0.6 ms (*n* = 13). This latency is consistent to that reported elsewhere when studying monosynaptic connections using optogenetics between two nuclei separated by a similar anatomic distance (Felix-Ortiz et al., 2013; Holloway et al., 2013). Again we used the rescue protocol to confirm a direct connection; that is, while TTX was effective at eliminating the light-evoked response, addition of the K^+^ channel blockers 4-AP and TEA (Cousin and Robinson, 2000; Petreanu et al., 2009) restored the response in the neuron (Fig. 16*D*), which was *post hoc* identified with scRT-PCR as expressing *Cart* mRNA (Fig. 16*E*).

### Kiss1^ARH^ neurons excite PVH and DMH neurons via glutamate release

**Figure 17.**
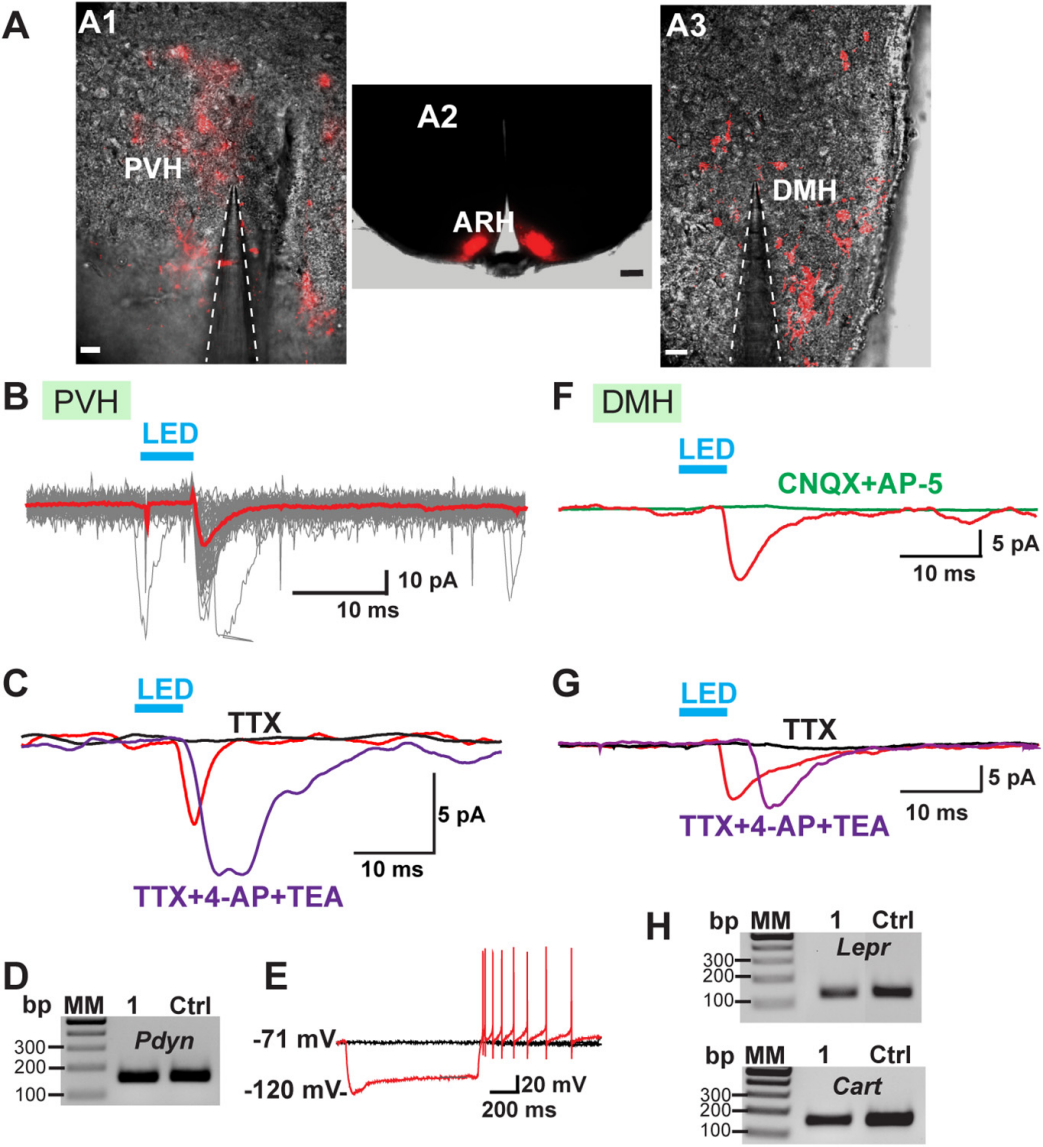
Photostimulation induced glutamate release from Kiss1^ARH^ fibers onto PVH and DMH neurons. ***A1***, ***A3***, High-power, overlay of DIC and epifluorescence (mCherry) images showing the Kiss1 terminals expressing mCherry (red) around a patched cell in PVH (***A1***) and DMH (***A3***). ***A2***, Low-power image of a coronal section through the ARH from Kiss1^Cre^ mouse that received dual injections of AAV-DIO-ChR2:mCherry. Scale bars: 40 μm (***A1***, ***A3***) and 200 μm (***A2***). ***B–E***, Blind patch recording in PVH neurons. ***B***, Fast glutamatergic current in voltage clamp V_hold_ = −60 mV following 5 ms of optostimulation. Red trace shows averaged response. Blue bar above recordings indicates LED stimulus. ***C***, The optogenetically evoked currents were blocked by TTX (1 μm, black trace), but recovered when the K^+^ blockers 4-AP (0.5 mm) and TEA (7.5 mm) were added to the bath (magenta trace). ***D***, A gel image of scRT-PCR of *Pdyn* expression in a recorded PVH neuron, which was documented in five responsive cells. MM, molecular markers; 1, the recorded PVH neuron; Ctrl, positive tissue control. ***E***, 75% of the Pdyn neurons exhibited a “sag” (h-current) with hyperpolarizing current injection and rebound burst firing in current clamp. ***F–H***, Blind patch recording in DMH neurons. Photostimulation before (red trace) and after blockade (green trace) following CNQX (10 μm) and AP5 (50 μm) application. ***G***, Photostimulation induced a fast EPSC following blue light stimulation (red trace). The response was abrogated in the presence of TTX (1 μm, black trace) but rescued with the addition of the K^+^ channel blockers 4-AP (0.5 mm) and TEA (7.5 mm; magenta trace). ***H***, Gel images of scRT-PCR of long form leptin receptor (*Lepr*) and *Cart* mRNA in the “rescued” DMH neuron in ***G***. MM, molecular markers; 1, the recorded DMH cell; Ctrl, positive tissue control.

We also did whole-cell patch recordings using a standard internal solution from PVH and DMH neurons adjacent to Kiss1-ChR2:mCherry fibers in OVX Kiss1^Cre:GFP^ mice that had received bilateral injections of AAV1-ChR2-mCherry into the ARH (Fig. 17*A1–A3*). Kiss1^ARH^ neurons are glutamatergic (Nestor et al., 2016; Qiu et al., 2016), and stimulation of Kiss1^ARH^ fibers in the PVH excited PVH neurons (Fig. 17*B*). We recorded EPSCs (Fig. 17*B*) from PVH neurons with a mean latency of 5.1 ± 0.4 ms (*n* = 23), which was antagonized by the glutamate ionotropic blockers CNQX (AMPA) and AP5 (NMDA; data not shown). Moreover, TTX abrogated the photostimulated postsynaptic inward current (Fig. 17*C*), but the inward current was rescued with the addition of the K^+^ channel blockers 4-AP and TEA to the bath (Cousin and Robinson, 2000; Petreanu et al., 2009), which is evidence for direct synaptic contact between Kiss1^ARH^ neurons and the postsynaptic responsive PVH neurons. These responsive neurons were located in the dorsomedial PVH with a resting membrane potential of −62.7 ± 3.5 mV, an input resistance of 1.1 ± 0.1 GΩ and a capacitance of 19.1 ± 0.9 pF (*n* = 21; Fig. 17*A1*). Ten of the neurons expressed *Vglut2* mRNA based on scRT-PCR identification, and five cells were found to express *Pdyn* mRNA, two of which also expressed *Vglut2* (Fig. 17*D*). Again, the majority (80%) of the responsive PVH neurons expressed an h-current, including four out of five *Pdyn* neurons exhibited a “sag” and rebound burst firing following a hyperpolarizing stimulus indicative of h and T-currents, respectively (Fig. 17*E*).

Parvocellular DMH neurons with an input resistance of 0.9 ± 0.2 GΩ, a capacitance of 15.8 ± 1.4 pF, and a RMP of −59.3 ± 4.0 mV (*n* = 10; Fig. 17*A3*) also exhibited fast glutamatergic synaptic input from Kiss1^ARH^ neurons; the inward current was blocked by the glutamate ionotropic receptor blockers AP5 (NMDA) and CNQX (AMPA; Fig. 17*F*). Once again, the latency to response onset was brief (5.8 ± 0.4, *n* = 10), suggesting a direct connection. EPSCs were also frequently seen in slices taken from castrated males where the cells displayed an average resting membrane potential of −56.6 ± 2.4 mV, input resistance of 1.1 ± 0.2 GΩ and a cell capacitance of 16.6 ± 1.2 pF (*n* = 12). The latency to response onset in all cells was 5.0 ± 0.4 ms (*n* = 22) similar to previous reports using optogenetics (Felix-Ortiz et al., 2013; Holloway et al., 2013). Furthermore, TTX abrogated the photostimulated postsynaptic inward current, but the current was rescued with the addition of the K^+^ channel blockers 4-AP and TEA (Cousin and Robinson, 2000; Petreanu et al., 2009), again providing biophysical evidence for direct synaptic contact between Kiss1^ARH^ neurons and parvocellular DMH neurons (Fig. 17*G*). Lastly, we harvested the optogenetically-stimulated neurons for scRT-PCR identification, and four cells expressed *Cart* mRNA with one of these neurons also expressing the long form leptin receptor (*Lepr*; Fig. 17*H*).

## Discussion

**Figure 18.**
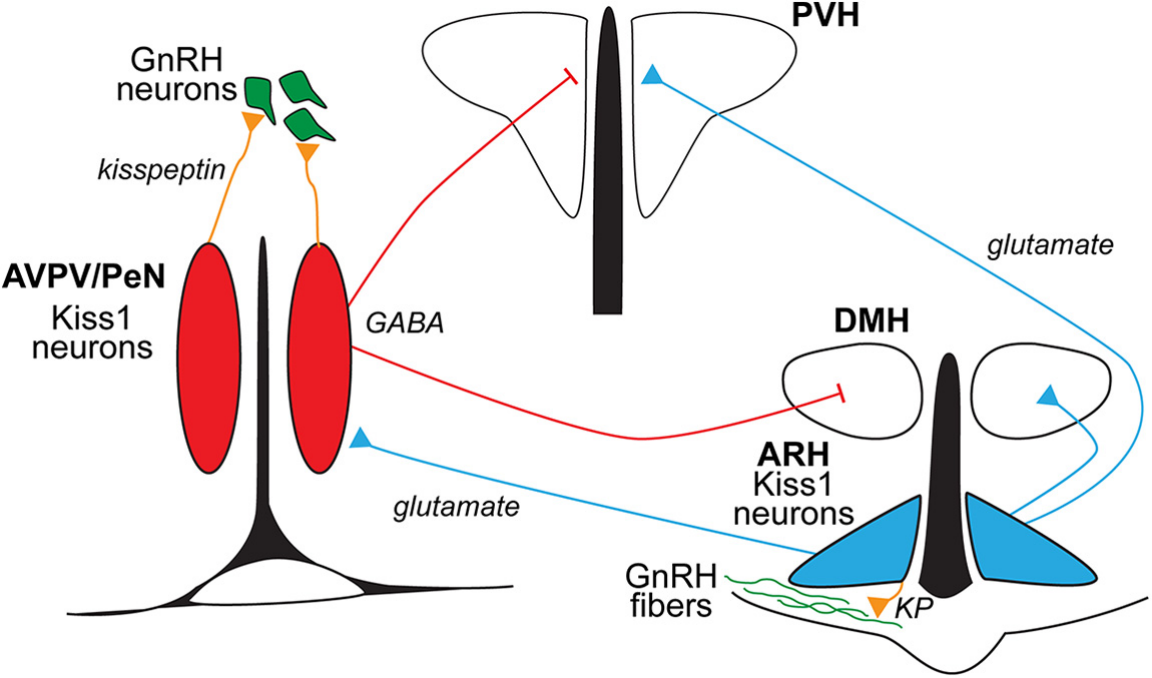
A highly schematic circuit diagram of anatomic and electrophysiological findings. The two populations of kisspeptin neurons (Kiss1 ^AVPV/Pen^ and Kiss1 ^ARH^) show overlapping but distinct projections to multiple hypothalamic nuclei including: DB, POA, SON, PVH, and DMH. More importantly, the two populations can use in addition to peptides their unique amino acid neurotransmitters, GABA for Kiss1^AVPV/Pen^ and glutamate for Kiss1^ARH^ neurons, to inhibit or excite parvocellular neurons in the PVH and DMH, areas that are known to control food intake and energy expenditure, respectively. These findings could be a general phenomenon for these two different populations of kisspeptin neurons. DB, diagonal band; POA, preoptic area; SON, supraoptic nucleus; PVH, paraventricular nucleus of the hypothalamus; DMH, dorsomedial hypothalamus.

Although Kiss1^ARH^ and Kiss1^AVPV/PeN^ neurons both send extensive projections to the ventro-lateral septum, BST and the PVH, they have distinct projection patterns to a number of hypothalamic areas including the VMPO, MnPO, MPA, DMH, and the ME. Functionally, we have found that activation of ChR2 expression in PVH or DMH nerve terminals from Kiss1^ARH^ neurons causes glutamate release and excitation of PVH and DMH neurons. In contrast, activation of ChR2 expression in nerve terminals from Kiss1^AVPV/PeN^ neurons causes hyperpolarization and inhibition of PVH and DMH neurons via GABA release. Therefore these different populations of Kiss1 neurons, in addition to their role to regulate reproduction, have the ability to differentially regulate nuclei, such as the PVH and DMH, that are important for neuroendocrine and autonomic regulation of numerous functions (Fig. 18; Lee et al., 2013a,b; Piñol et al., 2014; Shah et al., 2014).

### Kiss1 neuronal input to GnRH neurons

Currently we have shown in our Kiss1^Cre^ animal model, that Kiss1^AVPV/PeN^ neurons send projections toward GnRH neuronal somas and form close contact with the majority of these neurons, confirming previous data (Yip et al., 2015). This was expected, in large part because of previous findings that Kiss1^AVPV/PeN^ neurons directly activate GnRH neurons and appear to be responsible for the GnRH and LH surge at least in rodents (Clarkson et al., 2008; Qiu et al., 2016). Although, we did observe occasional close contacts between Kiss1^ARH^ fibers and GnRH cells and proximal dendrites in the POA, Kiss1^ARH^ neurons appear to primarily interact with GnRH nerve fibers and terminals in the ME area to regulate GnRH and subsequent LH pulsatility necessary for reproduction (Fox and Smith, 1985; O’Byrne et al., 1991; Mayer et al., 2010; Yip et al., 2015; Czieselsky et al., 2016; Qiu et al., 2016; Clarkson et al., 2017; Rønnekleiv et al., 2019; Voliotis et al., 2019; Liu et al., 2021).

### Kiss1 neurons and the SCN

Currently, we found little or no evidence for direct Kiss1 input to the central core part of the SCN in agreement with previous studies (Clarkson et al., 2009; Padilla et al., 2019). However, AVP neurons at the dorsal shell region of the SCN, and AVP efferent projections toward the SPa zone appeared to be contacted by both Kiss1^AVPV/Pen^ and Kiss1^ARH^ fiber-projections, which would indicate that Kiss1 neurons may modulate directly or indirectly a subgroup of AVP-positive SCN neurons and their output in the SPa zone. In support of such interactions, it was recently found that permanently silencing Kiss1^ARH^ neurons (with tetanus toxin) caused animals to become obese because of a diurnal shift in eating pattern, although their overall food consumption was not increased (Padilla et al., 2019). Kiss1^ARH^-silenced mice in comparison to controls ate significantly less during the dark phase with increased food intake during the light phase, which after four to eight weeks led to obesity. Kiss1^ARH^ silenced mice were also less active during the dark phase. Therefore, circadian disruption in feeding and reduced activity in the dark appeared to be contributing factors to increased obesity in Kiss1^ARH^ silenced mice. In this respect, earlier studies showing that mice fed a high-fat diet during the 12-h light phase gain more weight than mice fed this diet during the 12-h dark phase also point to the importance of feeding pattern on body weight (Arble et al., 2009). It has also been shown previously that rats subjected to hypocaloric, early morning restricted feeding for several months exhibited decreased number of immunoreactive AVP neurons and decreased AVP mRNA levels without major anatomic changes overall in the SCN, an indication that SCN AVP neurons may be involved in regulating diurnal feeding patterns (Andrade et al., 2004).

Kiss1^ARH^ neurons co-express kisspeptin, NKB, Dynorphin and glutamate (Goodman et al., 2007; Cravo et al., 2011; Nestor et al., 2016). Silencing of Kiss1^ARH^ neurons would eliminate release of all of these neuropeptides/neurotransmitters. Therefore, it is unclear by what cellular mechanisms Kiss1^ARH^ neurons influence the SCN clock to coordinate feeding pattern and activity with the dark phase. On the other hand, deletion of vGlut2 in Kiss1^ARH^ neurons eliminates glutamate release and leads to conditioned place preference for sucrose, but does not increase body weight on normal mouse chow (Qiu et al., 2018). Collectively, these findings suggest that Kiss1^ARH^ neurons are involved in regulating energy homeostasis, in part via glutamate release onto NPY/AgRP and POMC neurons and in part via Kiss1^ARH^ interaction with AVP neurons in the SCN to maintain diurnal feeding patterns (Qiu et al., 2018; Padilla et al., 2019).

### Kiss1 neurons and temperature regulation

The hypothalamus is a key region of the brain involved in the control of core body temperature (Tc) and energy homeostasis, in part via E2 regulation (Qiu et al., 2006; Roepke et al., 2010). Neurons within the ARH, including NPY/AgRP, POMC, and Kiss1 (KNDy) neurons, are essential for the estrogenic control of energy homeostasis, and Kiss1^ARH^ neurons may also be involved in temperature regulation (Rance et al., 2013; Padilla et al., 2018; Rønnekleiv et al., 2019). Within the POA, the VMPO expresses warm-sensitive neurons that respond within seconds to increased ambient temperature, and glutamatergic and GABAergic neurons within the MnPO are sensitive to either heat or cold exposure (Tan et al., 2016; Abbott and Saper, 2017; Morrison and Nakamura, 2019). Importantly, these POA neurons are part of neurocircuitries that help to maintain a relatively constant Tc. It is well known that postmenopausal women lacking E2 may experience hot flushes, a periodic sensation of intense heat that negatively affects their quality of life (Freedman et al., 1995; Freedman, 2014). Kiss1^ARH^ neurons are proposed to play a key role in causing hot flushes based on observations that these neurons projects to preoptic thermoregulatory areas that express NK3R, in particular the MnPO, and that ablation of Kiss1^ARH^ neurons partially blocks the effects of estrogens on thermoregulation (Krajewski et al., 2010). However, recently it was discovered that NK3R-expressing neurons in the MnPO are not activated by warm sensors in the skin and are not warm-sensitive neurons, although they play a role in reducing Tc (Krajewski-Hall et al., 2019). The authors concluded that KNDy neurons modulate thermosensory pathways indirectly for heat defense. Currently, we have shown that Kiss1^ARH^ neurons send extensive projections to the AVPV, MPO, and PeN nuclei, but have a very limited input to the temperature-sensitive neurons in the VMPO and MnPO. In contrast, Kiss1^AVPV/PeN^ neurons project extensively throughout the POA, including the VMPO and the MnPO regions. Therefore, our results support the idea of an indirect input from Kiss1^ARH^ neurons to POA warm-sensitive neurons, perhaps via activation of Kiss1^AVPV/PeN^ neurons (Qiu et al., 2016, 2018). Clearly, additional studies are needed to elucidate how each of the Kiss1 neuronal groups may be involved in modulating temperature-regulating circuitries.

### Kiss1 neurons and the PVH and SON

Interestingly, we have demonstrated that the PVH appears to receive about equal fiber-input from Kiss1^AVPV/PeN^ and Kiss1^ARH^ neurons. Using immunocytochemical staining for kisspeptin, several authors have documented that kisspeptin-positive fibers are located in the PVH; these studies, however, did not differentiate the origin of these fibers (Clarkson et al., 2009; Marraudino et al., 2017). The PVH is a complex, heterogeneous nucleus consisting of magnocellular and parvocellular neurons (Hallbeck et al., 2001; Stern, 2015). The neuroendocrine AVP and OT magnocellular neurons in the PVH and SON send projections to the posterior pituitary where AVP and OT are released into the circulation to regulate blood osmolality and milk ejection, respectively (Stern, 2015; Brown et al., 2020; Tasker et al., 2020). Currently, we have shown that neurons expressing either AVP or OT are among the PVH neurons receiving close-contact fiber-input from both Kiss1^AVPV/PeN^ and Kiss1^ARH^ neurons. However, we found that OT neurons mainly in the SON are contacted directly by Kiss1^AVPV/PeN^ neuronal fibers with few if any fibers contacting directly AVP-expressing SON neurons, and there is no apparent input to the SON from Kiss1^ARH^ neurons. These data are consistent with findings in the rat that Kiss1^AVPV/PeN^ neurons send extensive projections to the SON and SON perinuclear zone at the end of gestation to increase the activity of OT neurons at the time of parturition (Seymour et al., 2017).

The parvocellular PVH comprises different neuronal types, including neuroendocrine neurons (e.g., AVP) that project to the ME and regulate adrenocorticotropin hormone (ACTH) secretion from anterior pituitary corticotrophs (Verbalis et al., 1986). The parvocellular PVH also consists of preautonomic neurons (e.g., dynorphin, vGluT2, and OT) that send projections to brainstem and spinal cord areas and are important for autonomic functions (Hallbeck et al., 2001; Stern, 2001, 2015; Stocker et al., 2006; Rood and De Vries, 2011). Currently we have identified AVP-expressing and OT-expressing neurons within the different subregions of the PVH that receive Kiss1 fiber input as revealed by close contact (confocal) analysis of fibers originating from either or both Kiss1^AVPV/PeN^ and Kiss1^ARH^ neurons. While useful in circuit mapping, close contact analysis does not provide conclusive evidence of synaptic contact. Therefore, we followed up these experiments with CRACM using optogenetic stimulation of Kiss1^ARH^ and Kiss1^AVPV/PeN^ nerve-terminals to document that *Pdyn*-expressing and *Vglut2*-expressing PVH neurons in the medial parvocellular PVH receive direct excitatory input only from Kiss1^ARH^ neurons and direct inhibitory input only from Kiss1^AVPV/PeN^ neurons. Indeed, we have found that low frequency stimulation of fiber-input from Kiss1^AVPV/PeN^ neurons to PVH neurons caused inhibition via GABA release. Importantly, high-frequency stimulation also inhibited PVH neurons via the Gi,o-coupled GABA-B receptor blocked by CGP55845 rather than excitation via kisspeptin, which would also be released with high-frequency stimulation (Qiu et al., 2016). Also, it has been shown previously that PVH neurons do not express the Kiss1 receptor (GPR54; Herbison et al., 2010), findings supported by our current data. These parvocellular PVH neurons are known to be critically involved in appetite regulation (Chang et al., 2007; Garfield et al., 2015; Li et al., 2018); however, the specific circuitries have not been completely documented and need to be further investigated. Interestingly, PVH dynorphin-expressing and enkephalin-expressing neurons exhibit increased mRNA expression and peptides in response to increased dietary fat consumption, which is known to promote additional feeding, a phenomenon called “fat-induced hyperphagia” (Chang et al., 2007). Also, there is glutamatergic input from the ARH onto *Mc4r*-expressing PVH neurons, which has been found to regulate satiety (Fenselau et al., 2017). The glutamatergic ARH to PVH projection was thought not to be from Kiss1^ARH^ -expressing neurons based on the low response-rate following activation of Kiss1^ARH^ projections. In addition, stimulation of Kiss1^ARH^ neurons did not rapidly suppress feeding during the dark cycle (Fenselau et al., 2017). Therefore, although Kiss1 neurons have direct projections to and may regulate a number of neurons involved in feeding behavior including those expressing NPY/AgRP and POMC in the ARH and now dynorphin and vGluT2 in the PVH (current findings and Chang et al., 2007; Qiu et al., 2018), additional experiments are needed to more fully explore the role of the two populations of Kiss1 neurons in controlling energy metabolism.

### Kiss1 neurons and the DMH

Kisspeptin neurons and Kisspeptin fiber-input have been described in the DMH in a number of species including mouse, guinea pig, sheep, monkey and human (Franceschini et al., 2006; Clarkson et al., 2009; Hrabovszky et al., 2010; Lehman et al., 2010; Bosch et al., 2012). However, the origin and specific role of Kiss1 neuronal input to the DMH has not been investigated. The DMH neurons express cholecystokinin (CCK), NPY, CART, and LepR and have been implicated in temperature regulation, body weight regulation and brown adipose tissue (BAT) thermogenesis (Elias et al., 2000; Chen et al., 2004; Draper et al., 2010; Zhang et al., 2011; Tan et al., 2016; Morrison and Nakamura, 2019). Importantly, optogenetic stimulation *in vivo* of LepR neurons in the DMH, some of which also co-express CART or NPY, induces energy expenditure through action on sympathetic and BAT circuitries (Elias et al., 2001; Zhang et al., 2011; Lee et al., 2013b). Moreover, ablation of LepR from DMH neurons causes weight gain by reducing energy expenditure and locomotor activity (Rezai-Zadeh et al., 2014). Currently we have documented that Kiss1^ARH^ neurons project to DMH neurons and optogenetic activation of their fiber-terminals excites DMH neurons, including those expressing LepR and CART, via glutamate release. Therefore, the Kiss1^ARH^ neurons might be involved in regulating energy expenditure via direct actions on the DMH. In addition, temperature-sensitive POA neurons act directly on DMH neurons to regulate BAT activity. The inhibitory GABAergic output from the POA to DMH neurons leads to suppression of BAT thermogenesis during warm ambient conditions (Madden and Morrison, 2019). Currently we have found that Kiss1^AVPV/PeN^ neurons send extensive fiber-projections to the DMH, and optogenetic activation of these fibers induced an outward current via GABA release that inhibited DMH neurons. Therefore, Kiss1^AVPV/PeN^ neurons may be involved in suppressing BAT thermogenesis via action on DMH neurons during heat exposure and, thus, have a role in regulating energy expenditure as well as core body temperature. Interestingly some DMH cells displayed two sets of responses with distinct latencies, and a subset of recordings taken in Kiss1::Ai32 mice showed mixed GABA/Glutamate inputs (two out of eighteen responsive neurons). Therefore, Kiss1^AVPV^ and Kiss1^ARH^ neurons appear capable of projecting to the same neurons. While glutamatergic inputs with two different latencies might reflect direct and indirect inputs, dual GABAergic responses must be via two mono-synaptic inputs. Also, although many postsynaptic responses were seen in the DMH, the frequency of response was about half of what was recorded in the PVH. Therefore, further work needs to be done to characterize the DMH subpopulations targeted by Kiss1 fibers and focusing on specific DMH subpopulations may yield greater consistency in observing postsynaptic responses.

### Summary

It is well documented that hypothalamic Kiss1 neurons are necessary for pubertal development and adult reproduction (De Roux et al., 2003; Seminara et al., 2003; d’Anglemont de Tassigny et al., 2007; Moore et al., 2019). In addition, these neurons send inhibitory and excitatory projections to multiple brain regions involved in functions such as metabolism, energy expenditure and temperature regulation. Although, Kiss1^ARH^ neurons have been proposed to be involved in the regulation of core body temperature and may induce hot flash symptoms in mice (Padilla et al., 2018; Krajewski-Hall et al., 2019), Kiss1^AVPV/PeN^ neurons appear to have the most extensive direct projections to temperature-sensitive areas (current findings). Moreover, we have documented direct projections of Kiss1 neurons to parvocellular neurons in the PVH and DMH (Fig. 18), which are known to be involved in regulating food intake and energy expenditure, respectively. Clearly, further studies are needed to elucidate the mechanisms by which Kiss1 neurons affect these other homeostatic functions.

## Synthesis

Reviewing Editor: Quentin Pittman, University of Calgary Decisions are customarily a result of the Reviewing Editor and the peer reviewers coming together and discussing their recommendations until a consensus is reached. When revisions are invited, a fact-based synthesis statement explaining their decision and outlining what is needed to prepare a revision will be listed below. The following reviewer(s) agreed to reveal their identity: Michael Lehman.

This is a thorough analysis of hypothalamic neuronal projections from the two major populations of kisspeptin cells in the mouse brain, the AVPV/PEN and ARH, using viral based vectors that also incorporated channel-rhodopsin to allow for electrophysiological confirmation of contacts and interrogation of contacted neurons in the PVN and DMH. Overall, the work has been carefully carried out and provides an insightful descriptive anatomical analysis of the projection patterns from these two different populations.

However, the anatomical contribution, while solid, with compelling photomicrographs, is a somewhat incremental addition to knowledge in the field as anterograde viral tracing from the two kisspeptin populations has been performed before (Yip et al., 2015). Thus, the cell-specific inputs to VP and OT neurons in the PVN, and electrophysiological characterization of those inputs to PVN and DMH neurons which have not been previously reported are the particular unique contribution. However, there are a number of considerable deficiencies that must be addressed in a revision.

Anatomical issues:

1. Analyses of the % GnRH neurons receiving close contacts from Kiss1AVPV/PEN and Kiss1ARH fibers should be performed per animal and reported as the mean {plus minus} S.E.M. for each group. The same type of analysis (% cells with close contacts) should be applied to close contacts with VP and OT neurons in the SON and PVN (each subdivision). It might be easiest to summarize this information in Table format, along with mean number of GnRH, VP, OT neurons analyzed. Note that the journal encourages use of estimation statistics for any statistical analysis in this regard

2. Overall, the close contact super-resolution analysis is useful and informative, but definitive identification of synaptic inputs requires either co-labeling with synaptic markers (e.g., synaptophysin) or EM. The authors should note this caveat in the discussion. This is particularly relevant to the possibility of Kiss1ARH input to the dorsomedial shell of the SCN - it may be that effects of silencing Kiss1ARH neurons on diurnal feeding rhythms (brought up in the Discussion) may be mediated through indirect pathways rather than directly. Is there any evidence that SCN VP cells play a specific role in diurnal or circadian rhythms of food intake?

3. Were there any Kiss1AVPV/PEN contacts on GnRH cells located more rostrally than the POA, e.g., the medial septum (Fig. 2A, B) or diagonal band?

4. Since results from the anterograde tracing showed that Kiss1AVPV/PEN neurons project to PVN OT neurons, the focus on PDyn-expressing cells in the electrophysiological part of this study makes the overall paper seem a bit disjointed. It would have been good if the authors could have added analysis of inputs to PDyn cells to the anatomical studies

5. The authors seem to be considering only contacts with somata in describing connections. These postsynaptic cells have dendrites and they don’t appear to be able to see any such axo-dendritic contacts; thus they might be missing many of the inputs, especially excitatory ones onto dendrites. This is especially important where they report ‘absence’ of connections within a nucleus ( eg SCN or VMH). At least with the VMH it has been known for years (Millhouse) that synaptic contacts are in a dendritic ‘halo’ around the nucleus and very few extrinsic fibres penetrate to the interior. Perhaps the same is true of the SCN? This should at least be discussed. And here would have been an optimum place to do the ephys to see if there was a monosynaptic connection that they can’t see ( axo-dendritic) or perhaps the connection is onto an intervening interneuron.

6.The authors state in the abstract that CRACM was used to determine inputs to a number of brain regions. Again, this is a bit of an overstatement; while the authors did that for PVN and DMH, they only used anterograde tract-tracing (albeit with the same viral vector) for all other brain regions.

Electrophysiological issues:

1. The electrophysiological data are almost randomly chosen, with low n’s, lack of quantitation, inability to relate for the most part to phenotype (AVP, Dyn or OT) in the PVN and DMH. There is also lack of sufficient detail to judge many of the experiments ( where was the light directed- cell bodies? Terminal areas? What was its frequency?). The reviewers strongly advise that the electrophysiological results must be strengthened with greatly increased ‘n’ and suggest a focus on better describing the distribution of afferents to phenotypically or electrophysiologically identified neurons in the PVN and or DMH. Furthermore, key experiments must be reported (antagonists, test of the monosynaptic nature of the inputs) in more than an anecdotal nature.

2. The following details should be added to the electrophysiological data:

• Methods: Please indicate pulse duration and intensity of LED stimulation

• How many cells were recorded from in total to obtain the ones displaying these light-evoked PSCs. Somewhat related to this point, the authors state that they blindly recorded from neurons in the vicinity of ChR2-expressing fibers, which does not sound completely “blind”.

• Please indicate the number of neurons that were tested with TTX and 4-AP/TEA. It would also be useful to indicate concentrations used in the main text in addition to the figure legends.

• Please also comment on the substantial increase in PSC latency that can be seen in Figures 15 and 16 in the presence of TTX and potassium channel blockers.

• Please indicate how many neurons were tested for a GABA-B input. 1-The authors do not report any statistical significance in any experiment, thus to make any statements such that a response was abrogated, blocked or rescued, the authors should include bar graphs/data plots, p-values and indicate the appropriate statistical tests used where necessary. In its current form the evidence is unconvincing.

3. The authors note that Kiss1 AVPV/PeN neurons are GABAergic while Kiss1 ARC neurons are glutamatergic and cite Cravo et al. 2011 to support this statement. However, in this publication, Cravo et al. note that 20% of Kiss1 AVPV/PeN neurons coexpress vGlut2, a marker for glutamatergic neurons, and 50% of Kiss1 Arch neurons coexpress GAD-67, a marker for GABAergic neurons. It would be useful if the authors could comment on the proportion of evoked glutamatergic and GABAergic currents observed onto PVN or DMH neurons. The current number of ns is insufficient to determine or claim whether all PVN (n=11) or DMH (n=6) projecting kiss1 AVPV/PeN neurons are GABAergic and/or PVN (n=9) or DMH (n=4) projecting Kiss1 ARC neurons are glutamatergic.

4. Based on the molecular identity of the target PVN or DMH neuron (obtained using scRT-PCR) can the authors comment on the proportion of evoked Glut/GABA input these neurons receive from respective Kiss1 AVPV/PeN or ARC neurons?

5. In Fig. 15B-E, the authors used a high chloride internal solution to observe IPSCs, which in this case are inward currents. However, it does not appear that the authors used any ionotropic glutamate receptor blockers to eliminate the possibility of detecting overlapping EPSCs, which would also be inward currents. The authors should demonstrate that they are recording isolated GABAergic currents.

6. The numbers of neurons that characterized as Prodynorphin-expressing in the PVN and LepR-expressing in the DMH are very low (3 and 1, respectively). Hence, the statement “optogenetic stimulation of Kiss1 projections to PVH prodynorphin and DMH leptin receptor-expressing neurons” in the abstract is a bit of an overstatement.

7. The use of 4-AP/TEA to infer monosynaptic contacts is interesting. Please comment upon the much longer latency to reverse the TTX effect; how can one be certain there is not recruitment of an interneuron in the pathway as a function of the TEA/4-AP effect on both the extrinsic terminal and a possible interneuron, in effect requiring additional Ca++ buildup in two serial synapses? (especially for the GABA inputs)?

## Author Response

1

Synthesis of Reviews:

Computational Neuroscience Model Code Accessibility Comments for Author (Required):

N/A

Significance Statement Comments for Author (Required):

Significance statement goes well beyond the data in that it suggests finding actions onto neurons controlling food intake, energy expenditure and brown fat thermogenesis. None of your recorded neurons were functionally described; rather you found projection to neurons in areas controlling these functions which may suggests input to ......

Response: The Significance statement has been rewritten

Comments on the Visual Abstract for Author (Required):

None present although a depiction of the circuits and connections identified could be a useful addition

Response: We have depicted a simple circuit diagram for the readers (Fig. 18).

Synthesis Statement for Author (Required):

This is a thorough analysis of hypothalamic neuronal projections from the two major populations of kisspeptin cells in the mouse brain, the AVPV/PEN and ARH, using viral based vectors that also incorporated channel-rhodopsin to allow for electrophysiological confirmation of contacts and interrogation of contacted neurons in the PVN and DMH. Overall, the work has been carefully carried out and provides an insightful descriptive anatomical analysis of the projection patterns from these two different populations.

However, the anatomical contribution, while solid, with compelling photomicrographs, is a somewhat incremental addition to knowledge in the field as anterograde viral tracing from the two kisspeptin populations has been performed before (Yip et al., 2015). Thus, the cell-specific inputs to VP and OT neurons in the PVN, and electrophysiological characterization of those inputs to PVN and DMH neurons which have not been previously reported are the particular unique contribution. However, there are a number of considerable deficiencies that must be addressed in a revision.

Response: We thank the Reviewers and Editor for helpful comments, which we have addressed in the revised manuscript. However, we respectfully disagree that the anatomical contribution is an “incremental addition to knowledge.” The Yip et al 2015 paper is directed more towards inputs onto GnRH neurons and the interaction between the two different groups of Kiss1 neurons. Importantly, Yip at al. were using another Kiss1-Cre mice for cell-specific tract tracing. There are several Kiss1Cre animal models, which are different both as far as co-expression of the classical neurotransmitters, Kiss1Cre neuronal distribution and projections, which is now described in the Introduction. Therefore, we believe that the projection analysis of another Kiss1Cre mouse model is warranted and provides more than an incremental addition to knowledge.2

Anatomical issues:

1. Analyses of the % GnRH neurons receiving close contacts from Kiss1AVPV/PEN and

Kiss1ARH fibers should be performed per animal and reported as the mean {plus minus} S.E.M. for each group. The same type of analysis (% cells with close contacts) should be applied to close contacts with VP and OT neurons in the SON and PVN (each subdivision). It might be easiest to summarize this information in Table format, along with mean number of GnRH, VP,

OT neurons analyzed. Note that the journal encourages use of estimation statistics for any statistical analysis in this regard.

Response: The close contact onto GnRH, AVP and OT neurons from the two groups of Kiss1 neurons has been performed for each animal and reported as mean for each group.

Also, the confocal data are summarized in Table 2.

2. Overall, the close contact super-resolution analysis is useful and informative, but definitive identification of synaptic inputs requires either co-labeling with synaptic markers (e.g., synaptophysin) or EM. The authors should note this caveat in the discussion. This is particularly relevant to the possibility of Kiss1ARH input to the dorsomedial shell of the SCN - it may be that effects of silencing Kiss1ARH neurons on diurnal feeding rhythms (brought up in the Discussion) may be mediated through indirect pathways rather than directly. Is there any evidence that SCN VP cells play a specific role in diurnal or circadian rhythms of food intake?

Response: We are aware that close contact is not synaptic contact, and have now made this clear in the Discussion (lines 552-556). We have also included a more detailed discussion of the role of SCN AVP neurons in feeding (lines 491-494).

3. Were there any Kiss1AVPV/PEN contacts on GnRH cells located more rostrally than the

POA, e.g., the medial septum (Fig. 2A, B) or diagonal band?

Response: Yes, and we have included a more detailed description of the location of GnRH neurons used for close contact analysis in the Results (lines 301-308).

4. Since results from the anterograde tracing showed that Kiss1AVPV/PEN neurons project to

PVN OT neurons, the focus on PDyn-expressing cells in the electrophysiological part of this study makes the overall paper seem a bit disjointed. It would have been good if the authors could have added analysis of inputs to PDyn cells to the anatomical studies

Response: We did not directly focus on dynorphin neurons. We used optogenetics to activate fiber-terminals within the PVH and recorded from cells close to these fibers. The cells were harvested following the recording and we used different primer pairs and scPCR to identify the recorded neurons. Interestingly, we found that many of the recorded cells expressed prodynorphin mRNA, but not oxytocin or AVP. Initially, we had used immunocytochemistry to stain for mCherry fibers interaction with dynorphin cell bodies in the PVH. However, our antibody stained primarily fibers and only a few scattered cells in this region, although, we were able to detect cells in the ARH, SON and the striatum. It should be noted that most studies of pDyn cells in the PVH are based on studies using 3 pDyn-Cre animals (see Shah et. al., PNAS; 2014). Our experimental design has been explained more clearly in the Methods (lines 158-163)

5. The authors seem to be considering only contacts with somata in describing connections.

These postsynaptic cells have dendrites and they don’t appear to be able to see any such axodendritic contacts; thus they might be missing many of the inputs, especially excitatory ones onto dendrites. This is especially important where they report ‘absence’ of connections within a nucleus (eg SCN or VMH). At least with the VMH it has been known for years (Millhouse) that synaptic contacts are in a dendritic ‘halo’ around the nucleus and very few extrinsic fibres penetrate to the interior. Perhaps the same is true of the SCN? This should at least be discussed.

And here would have been an optimum place to do the ephys to see if there was a monosynaptic connection that they can’t see (axo-dendritic) or perhaps the connection is onto an intervening interneuron.

Response: We have included a new figure (Fig. 9C) showing close contact onto GnRH dendrites by Kiss1ARH fibers. However, based on Figs 4 & 8, the VMH is essentially devoid of Kiss1 fiber input whereas the PVH and DMH are strongly innervated.

The close contact analysis we have employed has been used by several laboratories. Still it has its limits, which we have enumerated, and is why we chose complementary electrophysiological experiments. We agree that analysis of “dendritic halo” inputs be useful in future experiments. Currently, we have included the possibility of indirect connections in the Discussion.

6.The authors state in the abstract that CRACM was used to determine inputs to a number of brain regions. Again, this is a bit of an overstatement; while the authors did that for PVN and DMH, they only used anterograde tract-tracing (albeit with the same viral vector) for all other brain regions.

Response: The statement has been modified.

Electrophysiological issues:

1. The electrophysiological data are almost randomly chosen, with low n’s, lack of quantitation, inability to relate for the most part to phenotype (AVP, Dyn or OT) in the PVN and DMH. There is also lack of sufficient detail to judge many of the experiments ( where was the light directed cell bodies? Terminal areas? What was its frequency?). The reviewers strongly advise that the electrophysiological results must be strengthened with greatly increased ‘n’ and suggest a focus on better describing the distribution of afferents to phenotypically or electrophysiologically identified neurons in the PVN and or DMH. Furthermore, key experiments must be reported (antagonists, test of the monosynaptic nature of the inputs) in more than an anecdotal nature.

Response: In order to address this concern we have brought in Dr. Martin Kelly (OHSU) as a co-author to aid in the significant expansion of the electrophysiological data included in this manuscript.

The fiber-input from the ARH and AVPV/PeN Kiss1 neurons to the PVH was the densest in the medial region, and therefore, we obtained more responsive neurons in that part of 4 the PVH. Note that the recordings were done blindly and the recorded cells identified post hoc with sc-RT-PCR. We did not have pDyn-GFP or OT-GFP or AVP-GFP mice to cross with our Kiss1-Cre mice, which would be needed for a more extensive analysis. Such studies can be done in future experiments. More experimental details have been added for clarification. However, we did include “rescue” analyses for monosynaptic input from both

Kiss1 neuronal groups to the PVH and DMH neurons, which was a major goal of the electrophysiology experiments.

2. The following details should be added to the electrophysiological data:

• Methods: Please indicate pulse duration and intensity of LED stimulation

These details have been added in the Methods (lines 221-224).

• How many cells were recorded from in total to obtain the ones displaying these light-evoked

PSCs. Somewhat related to this point, the authors state that they blindly recorded from neurons in the vicinity of ChR2-expressing fibers, which does not sound completely “blind”.

We tried to make it clear that we targeted the area with the highest fiber density. With this in mind our positive hits ranged from 39% in the PVH to 17% in the DMH. We have included this information in the Discussion (lines 599-601).

• Please indicate the number of neurons that were tested with TTX and 4-AP/TEA. It would also be useful to indicate concentrations used in the main text in addition to the figure legends.

We have added the concentrations in the figure legends, and we did the rescue experiments on 4 cells total and saw a consistent 2 fold (mean = 2.03) increase in the latency with very

little jitter. This data has been added to the Results (lines 397-400).

• Please also comment on the substantial increase in PSC latency that can be seen in Figures 15 and 16 in the presence of TTX and potassium channel blockers.

Because there is not the “huge” action potential driving calcium entry into the synaptic bouton, more ChR2 depolarizing events were needed to build up calcium to generate synaptic release. This is consistent with previous published data, which we have cited in the

Results (lines 392-394, 397-400, 419-421, 449-450).

• Please indicate how many neurons were tested for a GABA-B input. 1-The authors do not report any statistical significance in any experiment, thus to make any statements such that a response was abrogated, blocked or rescued, the authors should include bar graphs/data plots, p values and indicate the appropriate statistical tests used where necessary. In its current form the evidence is unconvincing.

We have included a bar graph summarizing the robust GABA-B blockade with CGP 55845 in four cells with the appropriate statistical test (new Fig. 15F).5

3. The authors note that Kiss1 AVPV/PeN neurons are GABAergic while Kiss1 ARC neurons are glutamatergic and cite Cravo et al. 2011 to support this statement. However, in this publication, Cravo et al. note that 20% of Kiss1 AVPV/PeN neurons coexpress vGlut2, a marker for glutamatergic neurons, and 50% of Kiss1 Arch neurons coexpress GAD-67, a marker for

GABAergic neurons. It would be useful if the authors could comment on the proportion of evoked glutamatergic and GABAergic currents observed onto PVN or DMH neurons. The current number of ns is insufficient to determine or claim whether all PVN (n=11) or DMH (n=6) projecting kiss1 AVPV/PeN neurons are GABAergic and/or PVN (n=9) or DMH (n=4) projecting Kiss1 ARC neurons are glutamatergic.

Response: Previously, we used the Cravo et al 2011 citation, however the current study uses a different Kiss1 animal model in which the AVPV/PeN neurons clearly express Vgat and release only GABA. Vglut2 is not expressed in these rostral Kiss1 neurons. Similarly, the

ARH Kiss1 neurons only express Vglut2 and release only glutamate. This information has been added with the appropriate refs. In addition, we did new experiments to confirm the published data (Results, lines 380-385). Moreover, we carefully document that no

GABAergic postsynaptic responses were recorded with opto-stimulation of Kiss1-ARH neurons and likewise no glutamatergic responses after opto-stimulation of Kiss1-AVPV neurons.

4. Based on the molecular identity of the target PVN or DMH neuron (obtained using scRTPCR) can the authors comment on the proportion of evoked Glut/GABA input these neurons receive from respective Kiss1 AVPV/PeN or ARC neurons?

Posthoc identification using scRT-PCR is not always possible, particularly if recordings go on for an extended period. We do not have sufficient identified cells to make any

declarative statements as to which PVH or DMH subpopulations are targeted by Kiss1 projections. To get at this question, future studies will require crossing Kiss1-Cre mice with a fluorophore expressing mouse (e.g. pdyn-GFP) to comment on the strength and content of the input.

In the newly added data we used Kiss2xAi32 mice, which have ChR2 expressed in both the

ARH and AVPV Kiss1 populations, to perform an unbiased sampling. However, we found that the hit rate was quite a bit lower compared to injected animals and not an efficient use of time or resources. Therefore, we cannot confidently state whether or not the PVH or

DMH receives more GABA or glutamate Kiss1 inputs. In the end, we have similar numbers of GABAergic and glutamatergic responses. However, based on recordings in slices from the same animal, optogenetically-evoked postsynaptic responses were more frequently observed in the PVH than the DMH.

5. In Fig. 15B-E, the authors used a high chloride internal solution to observe IPSCs, which in this case are inward currents. However, it does not appear that the authors used any ionotropic glutamate receptor blockers to eliminate the possibility of detecting overlapping EPSCs, which 6 would also be inward currents. The authors should demonstrate that they are recording isolated

GABAergic currents.

We have added/replaced Figures 15B-H to include recordings using the standard internal solution to illustrate the robust outward current with optogenetic stimulation. We think that it strengthens the results to show the GABA-A response (always blocked by picrotoxin) with two different internal solutions.

6. The numbers of neurons that characterized as Prodynorphin-expressing in the PVN and LepR expressing in the DMH are very low (3 and 1, respectively). Hence, the statement “optogenetic stimulation of Kiss1 projections to PVH prodynorphin and DMH leptin receptor-expressing neurons” in the abstract is a bit of an overstatement.

We have increased the numbers substantially (see the revised Figures 15, 16 and 17). We now have included 20 identified Vglut PVH neurons (10 with GABAergic input from the

AVPV/PeN Kiss1 neurons and 10 from the ARH Kiss1 neurons and subsets of 9 Pdyn divided between the two groups. In addition, we have identified 4 Cart DMH neurons divided between the two inputs. However, per the reviewer’s comment, we have modified the statement in the abstract.

7. The use of 4-AP/TEA to infer monosynaptic contacts is interesting. Please comment upon the much longer latency to reverse the TTX effect; how can one be certain there is not recruitment of an interneuron in the pathway as a function of the TEA/4-AP effect on both the extrinsic terminal and a possible interneuron, in effect requiring additional Ca++ buildup in two serial synapses? (especially for the GABA inputs)?

As noted by others (Holloway et al., J. Neurosci 2013; Felix-Ortiz et al., Neuron 2013), there is a need for calcium build up in the nerve terminal, but critically we see very little jitter, which would argue for a direct synaptic connection
